# High-resolution melting of multiple barcode amplicons for plant species authentication

**DOI:** 10.1016/j.foodcont.2019.05.022

**Published:** 2019-11

**Authors:** Nicolai Zederkopff Ballin, Jone Omar Onaindia, Hadeel Jawad, Rafael Fernandez-Carazo, Alain Maquet

**Affiliations:** European Commission, Joint Research Centre (JRC), Geel, Belgium

**Keywords:** Authentication, DNA, Food fraud, High-resolution melting, Plants, Species identification

## Abstract

In recent years, species identification in herbs has attracted considerable attention due to several cases of fraud; hence inexpensive high-throughput authentication methods are highly welcomed. Species authentication is often performed through DNA analysis and several specific regions (barcodes) are considered suitable. Each barcode (Bar) possesses different qualities in terms of universality and discrimination power. A multiplexed format where information can be extracted simultaneously from several barcode regions is seemingly appropriate to ensure the power of both universality and discrimination. In this approach, we amplified DNA from five different barcode regions in a multiplexed PCR format followed by high-resolution melting (HRM). This multiplexed Bar-HRM approach was first applied to plants spanning the plant kingdom and then gradually narrowing down the genetic variability within the Lamiaceae and the Solanaceae families to finally reach closely related cultivars. Universality was demonstrated through distinct melting profiles obtained for species originating from 29 different families spanning the angiosperms, gymnosperm, mosses, and liverwort (Marchantiophyta). Discrimination power was retained for species, sub-species, and a few cultivars through the application of multivariate statistics to the high-resolution melting profiles. This preliminary investigation has shown the potential to discriminate a vast amount of species within the whole plant kingdom. It requires no *a priori* knowledge of the species' DNA sequence and occurs in a closed system within 2.5 h at a reduced cost per sample compared to other DNA based approaches.

## Highlights

• A DNA profiling platform for species authentication throughout the plant kingdom.
• Distinct and reproducible melting profiles were obtained for all tested species.
• Universality was demonstrated across 29 families spanning the plant kingdom.
• Specificity was demonstrated for related species, sub-species, and cultivars.

## 1 Introduction

Authentication of plant species is important in a variety of different areas such as the trade of illegal and endangered species, herbal medicine, and food authentication, where one species is replaced with a cheaper one. Substitution of plant species in herbs is substantial with a prevalence of 50% in a recent investigation (Direction générale de la concurrence, 2018). Triggered by the increased awareness of food fraud, actions to ensure food authenticity in the European Union (EU, 2002) and the United States of America (FDA, 2011) have been implemented.

Authentication of plant species can be performed through microscopy (Matias et al., 2016), detection of metabolites (Wielogorska, Chevallier, Black, Galvin-King, Delêtre, Kelleher, et al., 2018), species-specific peptides (Li et al., 2018), and DNA based techniques (Bosmali, Ordoudi, Tsimidou, & Madesis, 2017). The most appropriate technology depends on the plant material to be analyzed, but DNA is often a good choice. DNA is ubiquitous, robust to degradation, different between species, and relatively conserved within species. DNA is often considered a secondary marker that can be detected through the targeted approache (Ballin & Laursen, 2019). For the targeted approach, several DNA markers exist, including satellites, insertions and deletions (InDels), single nucleotide polymorphisms (SNPs), and barcode regions. Barcode regions are particularly popular because of a high variability flanked by conserved regions suitable for primer design. Potentially, this allows discrimination of a large number of species using the same primer pair.

A number of PCR primers amplifying barcode regions have been published (Hollingsworth, Graham, & Little, 2011), each showing different degrees of universality and specificity (discrimination power) (Braukmann, Kuzmina, Sills, Zakharov, & Hebert, 2017; De Mattia et al., 2011; de Vere, Rich, Ford, Trinder, Long, Moore, et al., 2012). Lack of concurrent universality and specificity from Sanger sequencing of barcode regions (Staats, Arulandhu, Gravendeel, Holst-Jensen, Scholtens, Peelen, et al., 2016) has increased the focus on new advanced techniques. Simultaneous sequencing of several DNA regions has grown in popularity with several next-generation sequencing (NGS) platforms available (Hawkins, de Vere, Griffith, Ford, Allainguillaume, Hegarty, et al., 2015). However, the read length on the popular NGS machines are limited (Vincent, Derome, Boyle, Culley, & Charette, 2017), which is below the size of several of the barcode regions (Hollingsworth et al., 2011). Nonetheless, in principle, NGS allows superior universality and discrimination power compared to Sanger sequencing. However, NGS is time-consuming and requires expensive instrumentation and reagents, complicated bioinformatic pipelines, specialized users, and multiple manual preparation steps. These disadvantages have resulted in Sanger sequencing of barcode regions being one of the preferred analytical choices in plant species identification; albeit only one individual amplicon can be sequenced at a time.

Information reflecting the size and sequence of individual amplicons can be extracted with high-resolution melting (HRM). High-resolution melting of barcode regions (Bar-HRM) has been used to analyze amplicons from several regions including the *ITS2* region that discriminated 12 species from the genus of *Hippophae* (Liu, Xiang, Zhang, Lai, Xiong, Li, et al., 2018) and in a multiplexed (*ITS2*) reaction that discriminated nine species used for tea. The *psbA-trnH* region discriminated *Panax notoginseng* from six adulterant species (Tong, Jiang, Huang, Cui, & Yuan, 2014). The *rbcL* region discriminated three medicinal plants (Osathanunkul, Madesis, & de Boer, 2015) and six plants used for cooking oil production (Ganopoulos, Bazakos, Madesis, Kalaitzis, & Tsaftaris, 2013). The *trnL* region discriminated 13 species from the Leguminaceae family (Madesis, Ganopoulos, Anagnostis, & Tsaftaris, 2012) and five species used for fruit juice (Faria, Magalhães, Nunes, & Oliveira, 2013). The *matK* region was used to discriminate five species of *Crocus sativus* (Villa, Costa, Meira, Oliveira, & Mafra, 2016) and three different *Lavandula* species in lavender honey (Soares et al., 2018). A combination of the *rbcL* and *trnL* regions (two individual simplex reactions) was used to identify eight broomrape species (Rolland, Dupuy, Pelleray, & Delavault, 2016). In these examples, species discrimination or identification was achieved, but with an application limited to a small number of species. It was concluded in a thorough HRM investigation of barcode regions in plants that a barcode combination could provide an identification rate of 99% (Osathanunkul, Suwannapoom, Osathanunkul, Madesis, & de Boer, 2016). It was therefore interesting to explore the possibility to expand the technology and allow HRM to operate on several barcode amplicons from a multiplexed PCR reaction. Theoretically, this would render a concurrent universality and a high discrimination power. Multiplexed HRM profiles will inevitably be more complex than simplexed HRM curves. To embrace this complexity, multivariate data analysis applied to the high-resolution melting profiles would be an advantage (Ballin & Mikkelsen, 2016; Liu et al., 2018). This work investigated the possibility to discriminate species through amplification of five barcode regions coupled with high-resolution melting (multiplexed Bar-HRM) and multivariate data analysis. The ability of this profiling approach (Ballin & Laursen, 2019) to accomplish universality through the plant kingdom alongside the ability to distinguish closely related species was investigated. The application and potential of Bar-HRM in food, plants, and herbs are reviewed in several articles (Druml & Cichna-Markl, 2014; Simko, 2016; Sun, Li, Xiong, Zhao, & Chen, 2016) but the multi-barcode approach presented here is novel.

## 2 Material and method

### 2.1 Barcode regions

DNA barcode regions of various sizes (to avoid overlapping melting transitions) were selected and included the internal transcribed spacer 2 (*ITS2*), the non-coding spacer tRNA-His photosystem II protein (*psbA-trnH*), the ribulose-1,5-bisphosphate carboxylase/oxygenase large subunit (*rbcL*), and the transfer ribonucleic acid - Leucine (*trnL*) and its loop (*trnL* loop). Median amplicon sizes in plants range from 87 for the *trnL* (loop) to 654 for the *rbcL* (Hollingsworth et al., 2011). Table 1 shows the selected PCR primers.

**Table 1:** Details on the plant barcode regions, amplicons, and PCR primers.

DNA region	Organelle	Primer ID	Amplicon length range^a^	Primer sequence	Reference
*ITS2*	Nuclear ribosomal	ITS-u3	407–1630	CAWCGATGAAGAACGYAGC	Cheng et al. (2016)
		ITS-u4		RGTTTCTTTTCCTCCGCTTA	
*psbA-trnH*	Chloroplast	psbA3′f	226–934	GTTATGCATGAACGTAATGCTC	Sang, Crawford, and Stuessy (1997)
		trnHf		CGCGCATGGTGGATTCACAATCC	Tate and Simpson (2003)
*rbcL*	Chloroplast	rbcL a_f	654–654	ATGTCACCACAAACAGAGACTAAAGC	Kress and Erickson (2007)
		rbcL a_rev		GTAAAATCAAGTCCACCRCG	Ferri, Corradini, Ferrari, Santunione, Palazzoli, & Alu’ (2015)
*trnL* c, d	Chloroplast	trnL c	201–2114	CGAAATCGGTAGACGCTACG	Taberlet, Gielly, Pautou, and Bouvet (1991)
		trnL d		GGGGATAGAGGGACTTGAAC	
*trnL* g, h (loop)	Chloroplast	trnL g	51–135	GGGCAATCCTGAGCCAA	Taberlet, Coissac, Pompanon, Gielly, Miquel, Valentini, et al. (2007)
		trnL h		CCATTGAGTCTCTGCACCTATC	

a The amplicon length ranges include primer sites and were based on GeneBank sequences for land plants. The ranges should be considered estimates (Hollingsworth et al., 2011).

### 2.2 Plant material

**Fig. 1:**
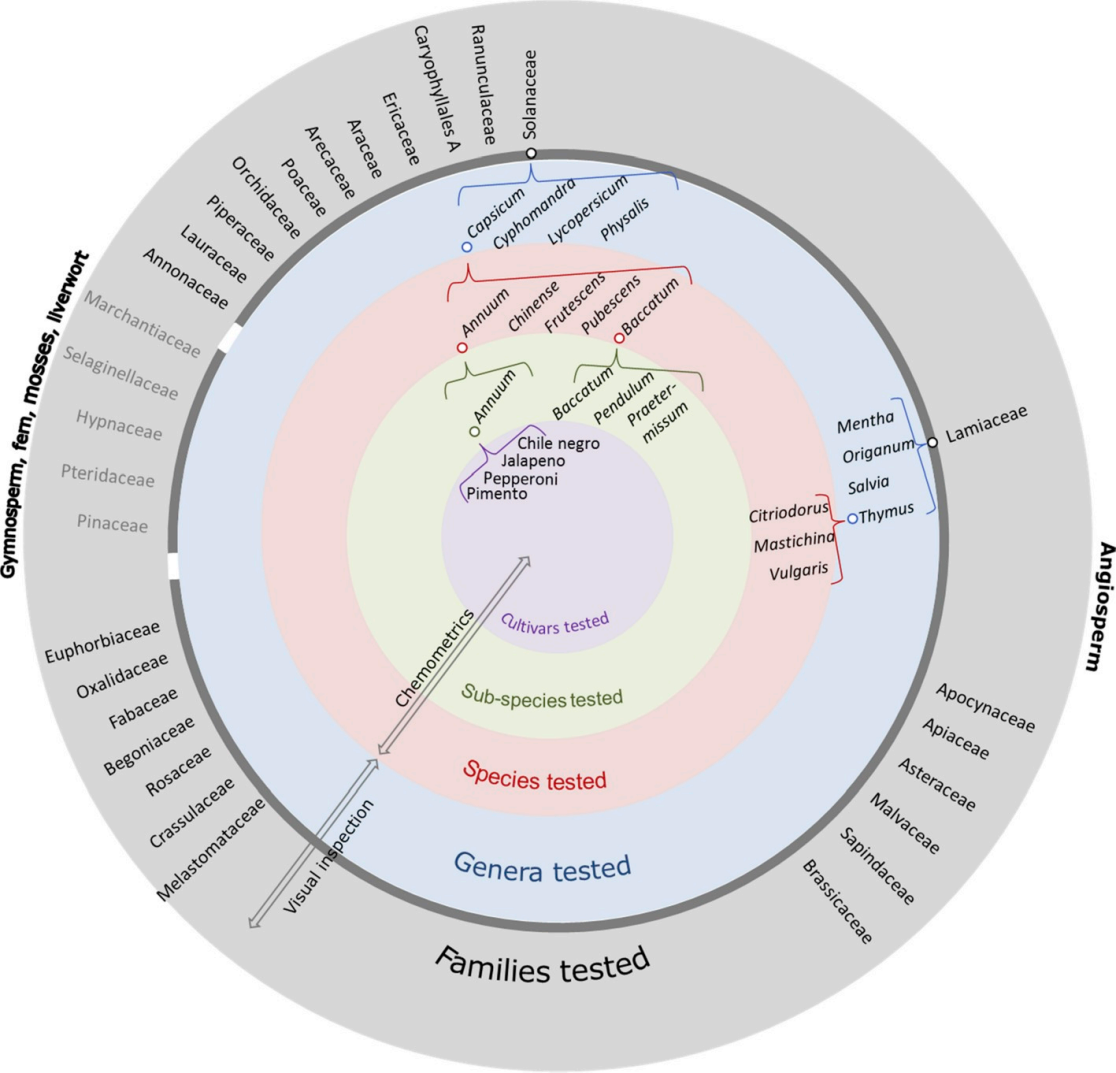
Families, genera, species, sub-species, cultivars, and their relationships are presented. The order of angiosperm families reflects their phylogenetic relationship from Annonaceae to Euphorbiaceae (Davies et al., 2004). Arrows indicate if examination of melting curves was performed through a visual or a chemometric approach.

From the Botanic Garden Meise in Belgium, fresh plant material from species representing different families was sampled using the following approach; from the angiosperms (Davies et al., 2004), plant orders that contained more than 1500 species were selected; resulting in 25 out of the total 42 orders. From the 25 orders, one species (if possible, a commercially important edible crop) from each of the largest families was sampled. Non-angiosperms included five species, one gymnosperm, one fern, two mosses, and one liverwort (Marchantiophyta). Also, *Trifolium alpestre* and three species representing different genera from the Lamiaceae and from the Solanaceae families were sampled. Finally, nine leaves were sampled from nine different wild plants within the same population of the species *Eupatorium cannabinum* native to the Botanic Garden of Meise. This material was sampled to investigate possible intra-population polymorphisms. The Leibniz Institute of Plant Genetics and Crop Plant Research (IPK) provided seeds from *Thymus* species and *Capsicum* species, sub-species, and cultivars. The Research and Economics (CREA), Research Centre for Forestry and Wood, Trento, Italy, provided *Thymus vulgaris* as dried material. The Agrifood Research and Technology Centre of Aragón, Spain, provided live *Thymus mastichina* plants, and the Crop Research Institute, Prague, Czech Republic, provided dried *Thymus citriodorus*. All plant materials were stored at −20 °C until DNA extractions were performed. Fig. 1 shows the relationships between families, genera, species, sub-species, and cultivars. Supplementary S1 contains detailed information on the plant materials and their usage.

### 2.3 DNA extraction

DNA from plant material was extracted with a semi-automated Maxwell 16 MDx Machine. In brief: 100–200 mg of fresh plant or 20–30 mg of dried plant material was placed in the round bottom 2 ml microcentrifuge tubes with two 5 mm stainless steel beads (Qiagen, Hilden, Germany). The material was beaten for 25 min with an oscillation of 50/s in a TissueLyser LT (Qiagen, Hilden, Germany). Hereafter, the protocol (TM473, revised 3/18) for the Maxwell RSC PureFood GMO and Authentication Kit (Promega, Madison, WI, USA) was used following 2 h incubation in an Eppendorf Thermomixer (Eppendorf, Hamburg, Germany) at 65 °C with 450 rpm. To investigate universality and discrimination of species from different genera, two technicians performed two DNA extractions for two days using kits with two different batch numbers. To study plant species from the same genus, sub-species and cultivars, two technicians performed six DNA extractions for six days using kits with three different batch numbers. To study repeatability, DNA was extracted from nine *T. vulgaris* samples on the same day. To study reproducibility, two technicians extracted DNA from six samples for six days using kits from three different batches. To study intra-population variability, DNA was extracted from nine individual plants from the species of *Eupatorium cannabinum* on the same day.

### 2.4 DNA purity and concentration

DNA purity (260 nm/280 nm ratio) was measured with the NanoDrop Lite™ (Thermo Scientific, Madison, WI, USA) instrument. Concentration was measured through dilution of 3 μl of extracted DNA diluted in 197 μl buffer using the Qubit 4 Fluorometer (Invitrogen, Singapore) and the broad range kit, Qubit dsDNA BR Assay kit (Invitrogen, Eugene, Orego, USA). New standards were prepared and measured for each batch of extracted DNA. DNA from all extractions was diluted with Nuclease-Free Water (Ambion, USA) and adjusted to 1 ng/μl. Diluted DNA was stored at −20 °C prior to analysis. Yield and purity (maximum and minimum) were recorded for each species or species group. As six or more determinations were performed, the standard deviation (std.) was calculated.

### 2.5 Multiplexed Bar-HRM

**Fig. 2:**
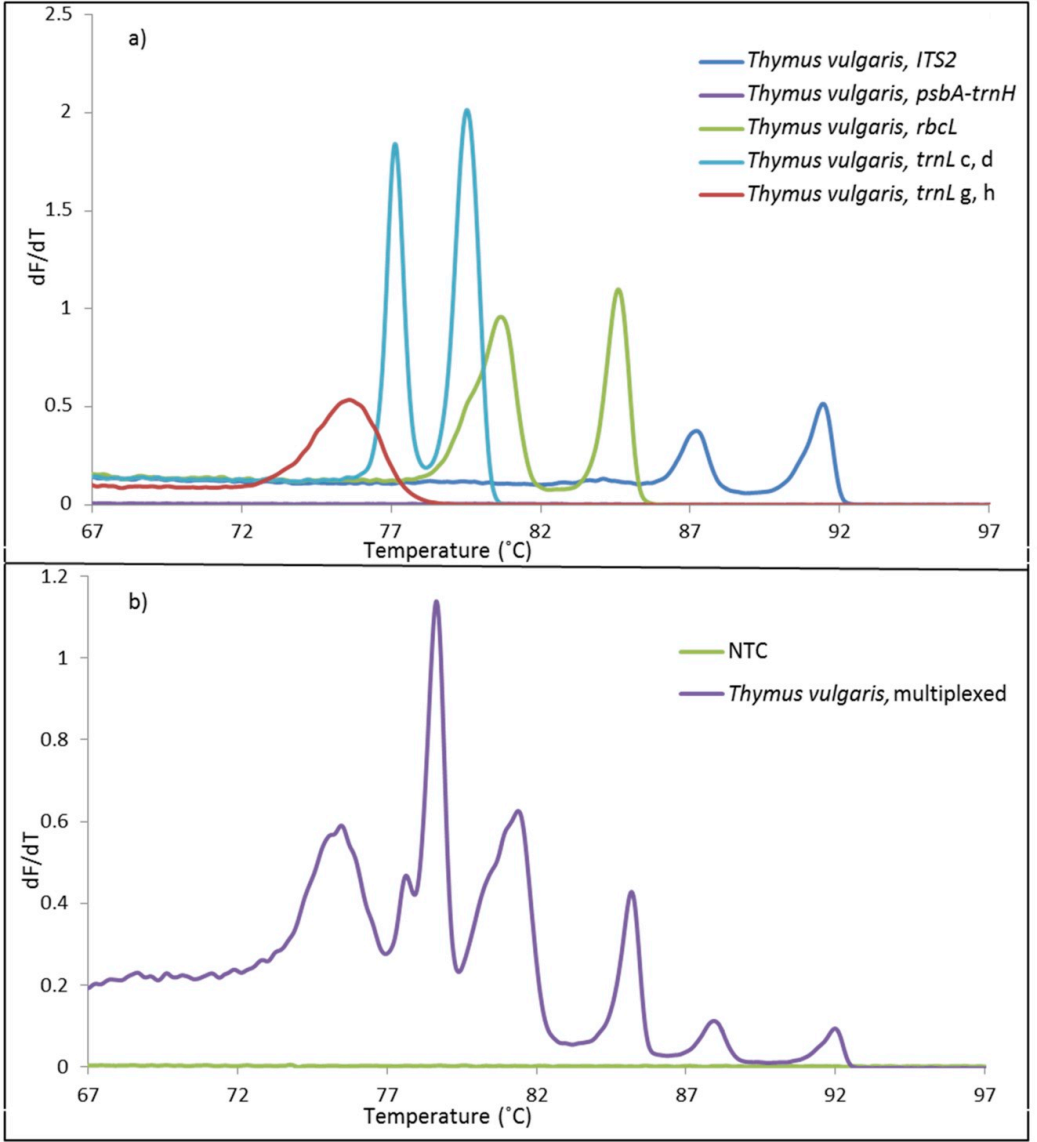
Panel a) Simplexed melting curves from *Thymus vulgaris*. Panel b) Multiplexed melting profiles from *T. vulgaris*. For clarity, only the average curve or profile of a duplicate analysis is presented. NTC: Non-template control.

**Fig. 3:**
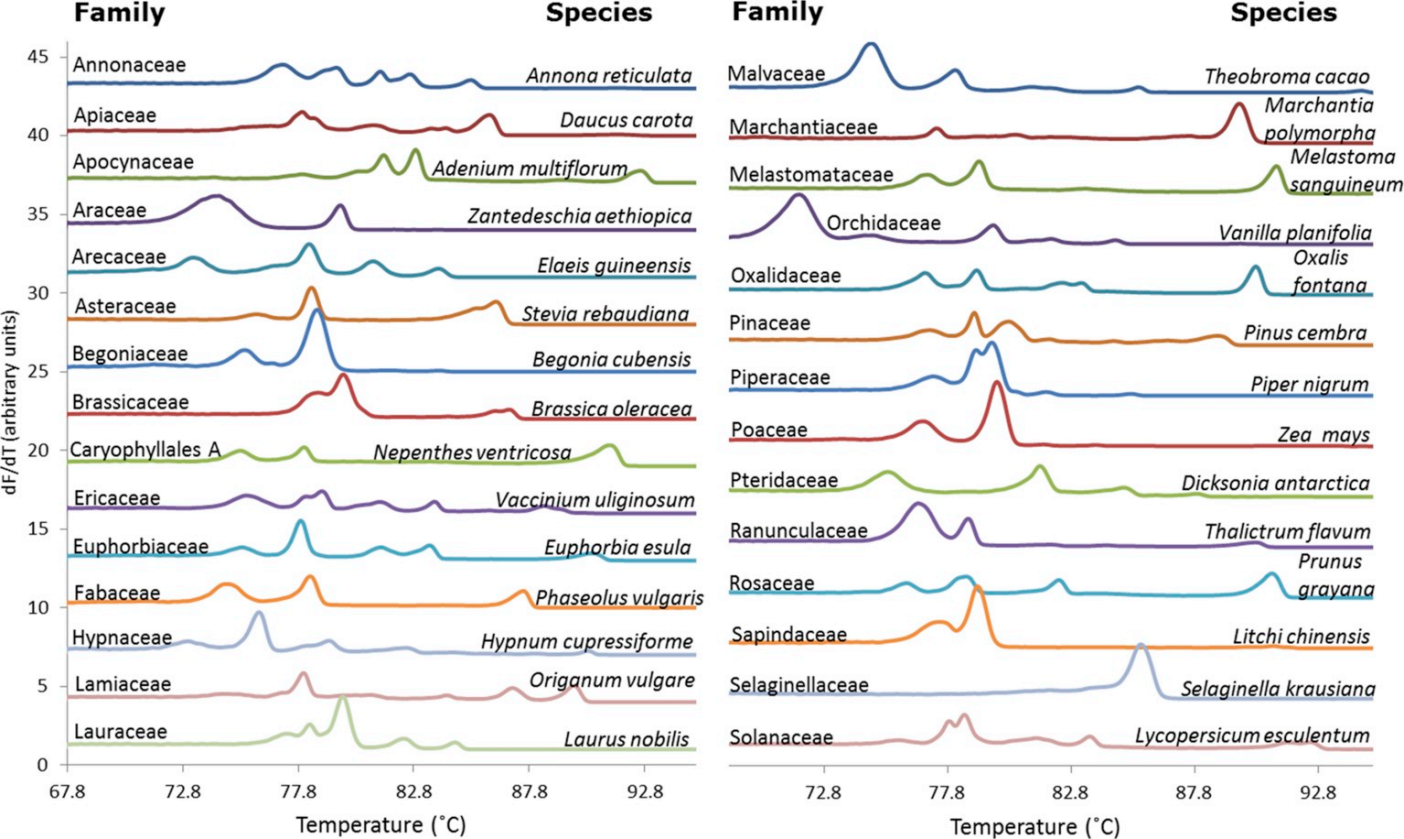
Multiplexed melting profiles from 29 plant species representing 29 families. For clarity, only the average profile of a triplicate analysis for each species is presented. See Supplementary S4, for the full sized melting profiles.

**Fig. 4:**
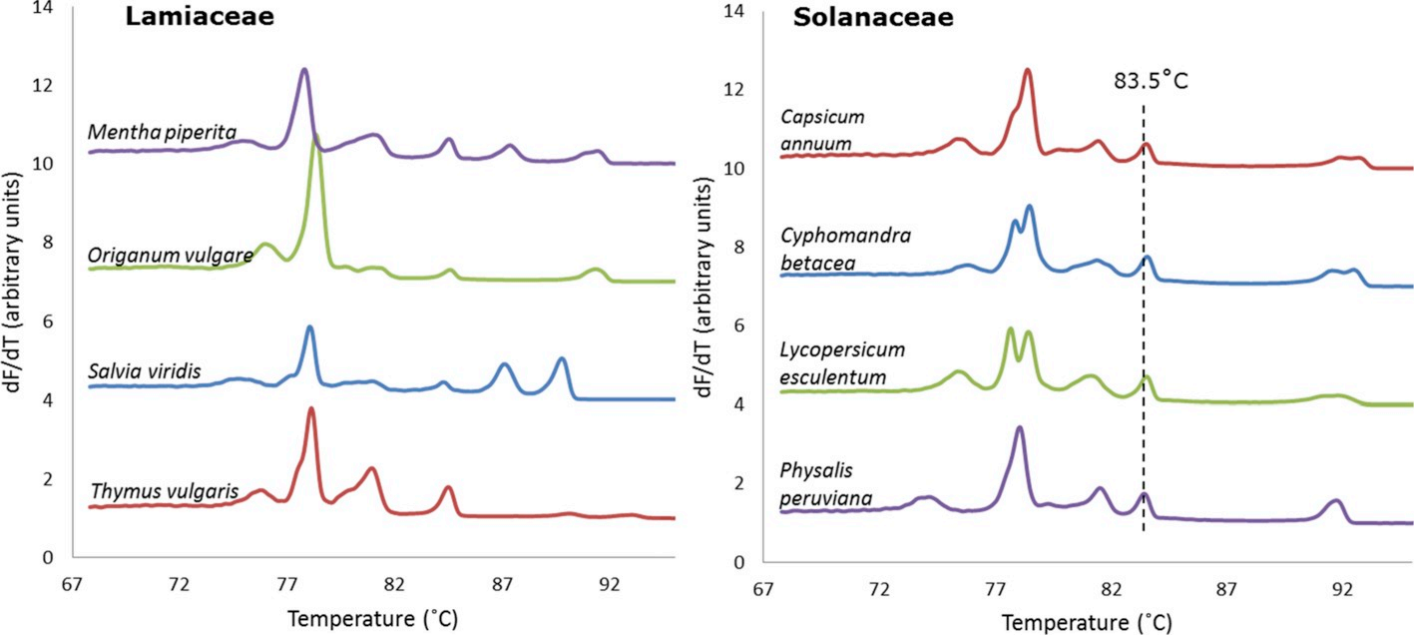
Multiplexed melting profiles of species from four plant genera within each of the Lamiaceae and Solanaceae families. For clarity, only the average profile of a triplicate analysis for each species is presented.

**Fig. 5:**
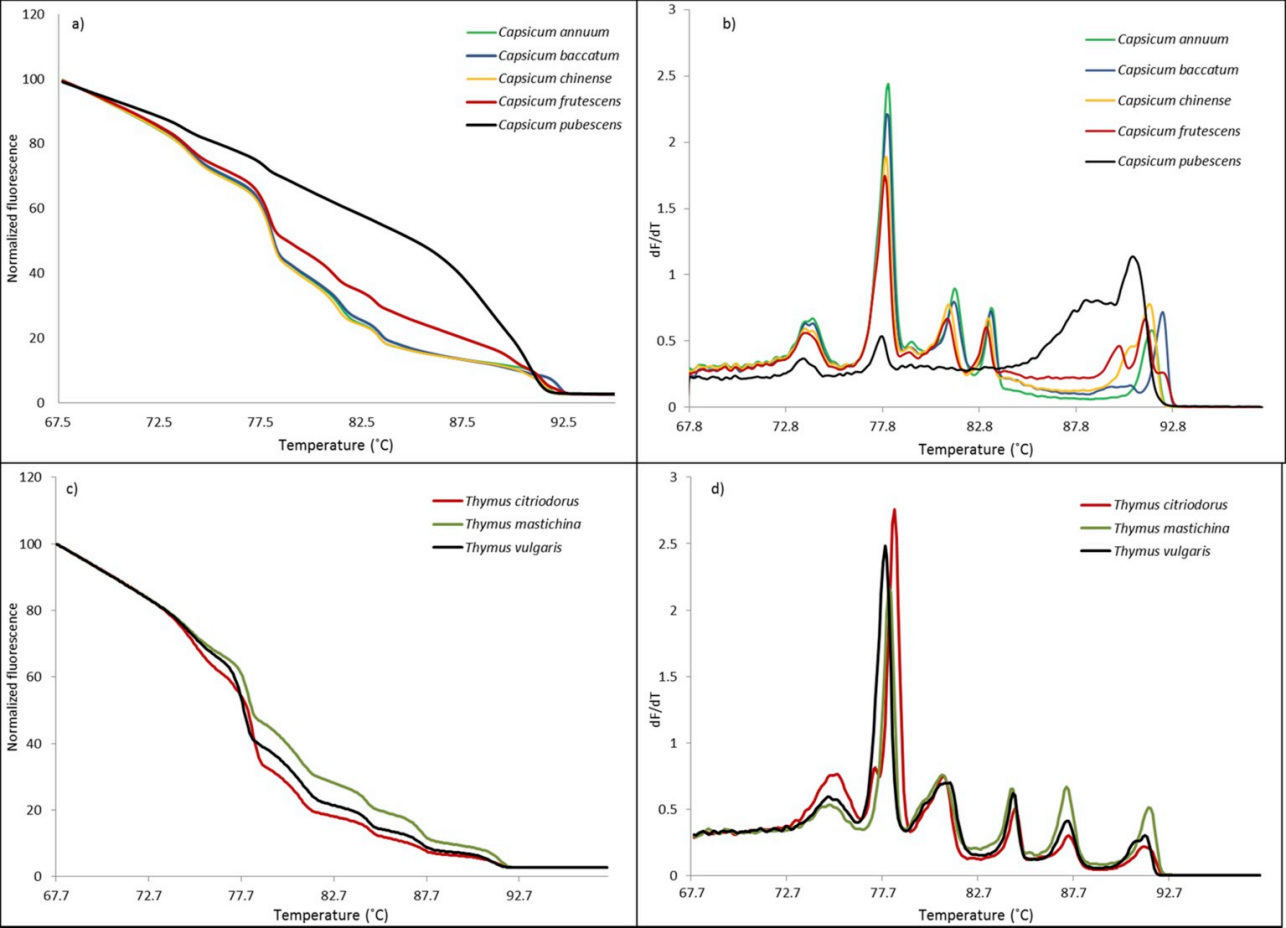
Panel a) and Panel b) Multiplexed high-resolution melting and melting profiles, respectively, of five *Capsicum* species; Panel c) and Panel d) Multiplexed high-resolution melting and melting profiles, respectively, of three *Thymus* species. For clarity, only the average profile of a triplicate analysis for each species is presented.

The PCR was carried out in a Rotor-Gene Q 6 plex (Qiagen, Hilden, Germany) instrument with the 72-rotor carrying 0.1 ml tubes (Qiagen, Hilden, Germany). The instrument was equipped with the Rotor-Gene Q Software 2.3.1 (build 49). It was important to optimize the PCR conditions to enable the display of a multiplexed melting profile that contained balanced information from the different barcode regions in different species. *Thymus vulgaris* was used for optimization and checked against *Capsicum* species to confirm that the primer pairs succeeded in producing melting profiles in other species under the same conditions. Different combinations of primer pair concentrations were mixed and tested in the multiplexed Bar-HRM approach to increase the intensity of individual melting transitions while avoiding amplification of the non-template control. When changes in individual primer pair concentrations did not further increase the intensity of individual melting transitions, other parameters were optimized, including EvaGreen and DNA concentrations, changes in annealing and extension temperature, and a different touchdown and long-range protocols (data not shown). The final conditions were as follows; the multiplexed PCR reaction mixture consisted of a volume of 25 μl that contained 12.5 μl AmpliTaq Gold™ 360 Mastermix (Thermofisher, Foster City CA, USA), 5.15 ng DNA, 1.35 μl EvaGreen^®^ Dye in water (Biotium, Inc. Fremont, CA, USA), and 6 μl of a primer mix consisting of the following concentrations: 6.1 μM *ITS2*, 1.2 μM *psbA-trnH*, 6.1 μM *rbcL*, 1.2 μM *trnL* c, d, 5.5 μM *trnL* g, h. For simplex reactions, 1 μl of a primer mix (10 μM) and 5 μl water was used. Primers were HPLC purified and shipped in a concentration of 100 μM from Invitrogen (ThermoFisher Scientific). PCR conditions: 95 °C for 10 min following 30 cycles of 95 °C for 15 s, 58 °C for 12 s, 72 °C for 30 s, and a final elongation step of 10 min at 72 °C. High-resolution melting was performed from 66 °C to 99 °C as an extension to the PCR program and consisted of 0.1 temperature increments for every 2 s. The gain was automatically optimized in all tubes on the HRM channel operating at an excitation wavelength of 460 nm and detection at 510 nm. The PCR-HRM program lasted 146 min. For each DNA extraction, one PCR-HRM experiment was set up. Duplicate analyses were performed for the evaluation and comparison of simplex and multiplexed PCR reactions. In all other PCR-HRM analysis, triplicate analyses were performed on each DNA extraction. For clarity, the average of replicates is presented in the melting profiles in Fig. 2, Fig. 3, Fig. 4, Fig. 5, Fig. 7, Fig. 8.

In *Thymus vulgaris*, the number of melting transitions in individual simplexed melting curves was analyzed *in silico* with the web-based uMELT software https://www.dna.utah.edu/umelt/umelt.html (Dwight, Palais, & Wittwer, 2011) and compared with the experimental curves.

### 2.6 Multivariate data analysis

Normalized HRM data from the Rotor-Gene Q software were exported as Excel files and imported to the Unscrambler®X software v10.5 (CAMO Software, Trondheim, Norway). Normalization was performed through scaling to a line of best fit with the highest fluorescence values set at 100 and the lowest value set at zero. Regions before and after the melting transition were selected to calculate the average fluorescence and slope and then applied in the normalization (Reja et al., 2010). In a second step, the first derivative of all the high-resolution melting profiles was carried out using Savitzky-Golay, polynomial order 2. Principal Component Analysis (PCA) was used to differentiate melting profiles of *Capsicum* and *Thymus* species and cultivars. The PCA models were created by mean centering the data, weights were fixed, and cross-validation (leave-one-out method, with NIPALS algorithm) was applied. The first derivative of the high-resolution melting profile was carried out to obtain the melting profile; four principal components (PCs) were required. PCA score plots visualized patterns in the sample distribution. Two *Capsicum* groups were inter-clustered in the PCA, and no clear separation could be observed; Partial Least Square - Discriminant Analysis (PLS-DA) was therefore applied (Tauler, 2002). The optimal number of latent variables was estimated by cross-validation, and a full validation with the leave-one-out method and Kernel PLS algorithm was applied. For *C. annuum* and *C. Chinense*, a dummy variable of 1 and 0, respectively, was applied.

## 3 Results and discussion

### 3.1 DNA extraction

For unknown reasons, DNA extraction from the species of *Crassula ovata* from the Crassulaceae family failed on both attempts. This sample was, therefore, excluded from the investigation. DNA from all other species was extracted. DNA yield and purity varied considerably due to the large variety of species subjected to extraction. Species (i.e. *Adenium multiflorum* (succulent plant), *Annona reticulata* (custard apple), *Begonia cubensis* (Cuban holly), *Brassica oleracea* (cabbage), *Daucus carota* (wild carrot), *Dicksonia antarctica* (soft tree fern), *Elaeis guineensis* (African oil palm), *Euphorbia esula* (green spurge), *Hypnum cupressiforme* (hypnum moss), *Laurus nobili* (tree or large shrub), *Litchi chinensis* (lychee), *Lycopersicum esculentum* (tomato), *Marchantia polymorpha* (common liverwort), *Melastoma sanguineum* (red melastome), *Nepenthes ventricosa* (pitcher plant), *Origanum vulgare* (oregano), *Oxalis fontana* (sourgrass), *Phaseolus vulgaris* (common bean), *Pinus cembra* (Swiss pine), *Piper nigrum* (black pepper), *Prunus grayana* (Japanese bird cherry), *Selaginella krausiana* (clubmoss), *Stevia rebaudiana* (candyleaf), *Thalictrum flavum* (common meadow-rue), *Theobroma cacao* (cacao), *Vaccinium uliginosum* (blueberry), *Vanilla planifolia* (vanilla), *Zantedeschia aethiopica* (calla lily), *Zea mays* (corn)) intended for the investigation of universality (two extractions) reached a DNA concentration between 1 ng/μl and 95 ng/μl with a purity between 1.34 and 2.0. The low DNA purity of 1.34 was obtained from the species of *Melastoma sanguineum* showing purities below 1.50 in both extractions. On average, all other species used for universality reached a DNA purity of above 1.50. DNA extracted from *Capsicum* species reached a DNA purity of between 1.38 and 1.88, with an average of 1.67 (std. 0.11). DNA extracted from *Thymus* species reached a DNA purity of between 1.42 and 2.0, with an average of 1.79 (std. 0.11). The nine *T. vulgaris* samples used for the repeatability study reached a DNA concentration of between 11.1 ng/μl and 22.3 ng/μl, with a purity of between 1.86 and 1.98 and an average of 1.9 (std. 0.04). DNA extractions for reproducibility (i.e. *Thymus vulgaris*) reached a DNA concentration of between 7.6 ng/μl and 22.3 ng/μl with a purity of between 1.70 and 1.86 and an average of 1.8 (std. 0.07). DNA extracted from the species of *Eupatorium cannabinum* used for the population study reached a concentration of between 28 ng/μl and 49 ng/μl, with a purity of between 1.46 and 1.64 and an average of 1.54 (std. 0.06). Differences in purity might influence individual melting profiles (Section 3.8).

### 3.2 Evaluation and comparing simplexed and multiplexed melting results

Primer pairs for each of the barcode regions were tested in simplex and multiplex reactions targeting *T. vulgaris*. The first derivatives are displayed in Fig. 2. The DNA amplicons comprising different DNA sequences and sizes displayed different Tm's, except for the *psbA-trnH* region that failed to amplify. This prompted us to investigate if the *psbA-trnH* region would amplify in other species. The amplification of the *psbA-trnH* region in other species, including *C. annuum* and *Trifolium alpestre* confirmed the usefulness of these primers (Supplementary S2). It is not surprising that a primer pair fails in some species as a result of a primer-template mismatch – no primer pair is fully universal. Another aspect to consider is the potential inhibition of individual primers that would result in a multiplexed profile that differed from a simple combination of the individual simplexed melting curves. This is easily recognized in Fig. 2 where the intensity of melting transitions from all amplified barcode regions has decreased in the multiplexed HRM profile compared to the simplexed curves. Fortunately, the multiplexed melting transitions mimic the combined simplexed reactions, enabling annotation of the multiplexed melting transitions to the individual barcode regions.

Fig. 2, Panel a, shows one melting transition from the amplification of the *trnL* g, h region, two melting transitions from the *trnL* c, d region, and a multi-step melting transition from the *ITS2* and the *rbcL* regions. Two or more melting transitions from a simplexed reaction could result from unspecific amplification, primer dimers, or a multi-step melting transition of the amplicon. An *in silico* melting analysis was therefore performed to ensure that the theoretical melting transitions of the amplicons matched the experimental ones. Supplementary S3 compares the *in silico* uMELT curves with the experimental melting curves. The *in silico* one-step melting of the *trnL* g, h amplicon, the two-step melting transitions of the *trnL* c, d amplicon, and the multi-step melting transition from the *ITS2* and *rbcL* amplicons agreed with the experimental melting transitions.

Fortunately, a dual or a multi-step melting transition could have a positive impact on the discrimination power as single mutations or small size differences have a higher influence relative to the sequence that melts. Basically, a decreasing amplicon size will increase the impact of single mutations on the melting curve (Słomka, Sobalska-Kwapis, Wachulec, Bartosz, & Strapagiel, 2017).

### 3.3 Universality

Twenty-nine plant species representing 29 families spanning the angiosperms and four plant species representing a gymnosperm, fern, mosses, and liverwort were analyzed to investigate the approach's universality across the plant kingdom. DNA from each species was subjected to the multiplexed Bar-HRM approach. The primers used for the individual barcode regions are generally much conserved, ensuring a PCR success over 90% (Cheng et al., 2016; Kress, Wurdack, Zimmer, Weigt, & Janzen, 2005; Taberlet et al., 2007). It was therefore not surprising to observe that our mixture of five primer pairs amplified and displayed distinct melting profiles from species across all tested families, Fig. 3. All melting profiles showed a complex structure (≥2 melting transitions) except for a moss species from the family of Selaginellaceae that only provided one major melting transition, which will possess limited discrimination power for genetically close samples. The general diverse melting profiles reflect the large genetic difference among the analyzed species. Melting profiles were displayed along a 22.8 °C range, starting from 71.8 °C in *Vanilla planifolia* to 94.6 °C in *Theobroma cacao*. Full-sized melting profiles are presented in Supplementary S4.

### 3.4 Discrimination of plant species from different genera within the Lamiaceae and the Solanaceae families

Four genera from each of the two families of Lamiaceae and Solanaceae were represented by the species, *Mentha piperita* (peppermint), *Origanum vulgare* (oregano), *Salvia viridis* (blue sage), *Thymus vulgaris* (thyme), and *Capsicum annuum* (pepper/chile pepper), *Cyphomandra betacea* (tamarillo), *Lycopersicum esculentum* (tomato), *Physalis peruviana* (Cape gooseberry), respectively. Melting curves from these eight species are presented in Fig. 4. These species are genetically less divergent compared to the species representing a large number of families along the plant kingdom (Fig. 3), as reflected in less divergent melting profiles. In the low temperature range, for example, there were similarities for the Lamiaceae species. The species from the Solanaceae family have a common melting transition at 83.5 °C. Nonetheless, the melting profile of each species is still unique.

### 3.5 Discrimination of related plant species from the same genus

The five species *C. annuum*, *C. baccatum, C. chinense*, *C. frutescens*, and *C. pubescens* from the *Capsicum* genus and the three species *T. citriodorus*, *T. mastichina*, and *T. vulgaris* from the *Thymus* genus were analyzed to investigate the discrimination power when the genetic difference between species was decreased, Fig. 5. The *C. baccatum, C. frutescens*, and *C. pubescens* melting profiles were visually easily discriminated; Fig. 5, Panel b. *Capsicum pubescens* differed significantly from the other *Capsicum* species in the lack of melting transitions of around 81 °C and 83 °C in addition to the extra melting transitions of around 88 °C. *Capsicum baccatum* and *C. frutescens* can be discriminated in the melting area of between 89 °C and 93 °C. *Capsicum annuum* and *C. chinense* were more difficult to discriminate using the melting profiles, and multivariate data analysis of the high-resolution melting profiles (Fig. 5, Panel a) was introduced to avoid a subjective conclusion. Besides introducing objectivity in the reporting of results, multivariate data analysis introduces a potential automated procedure that can be used for species identification between analytical series.

**Fig. 6:**
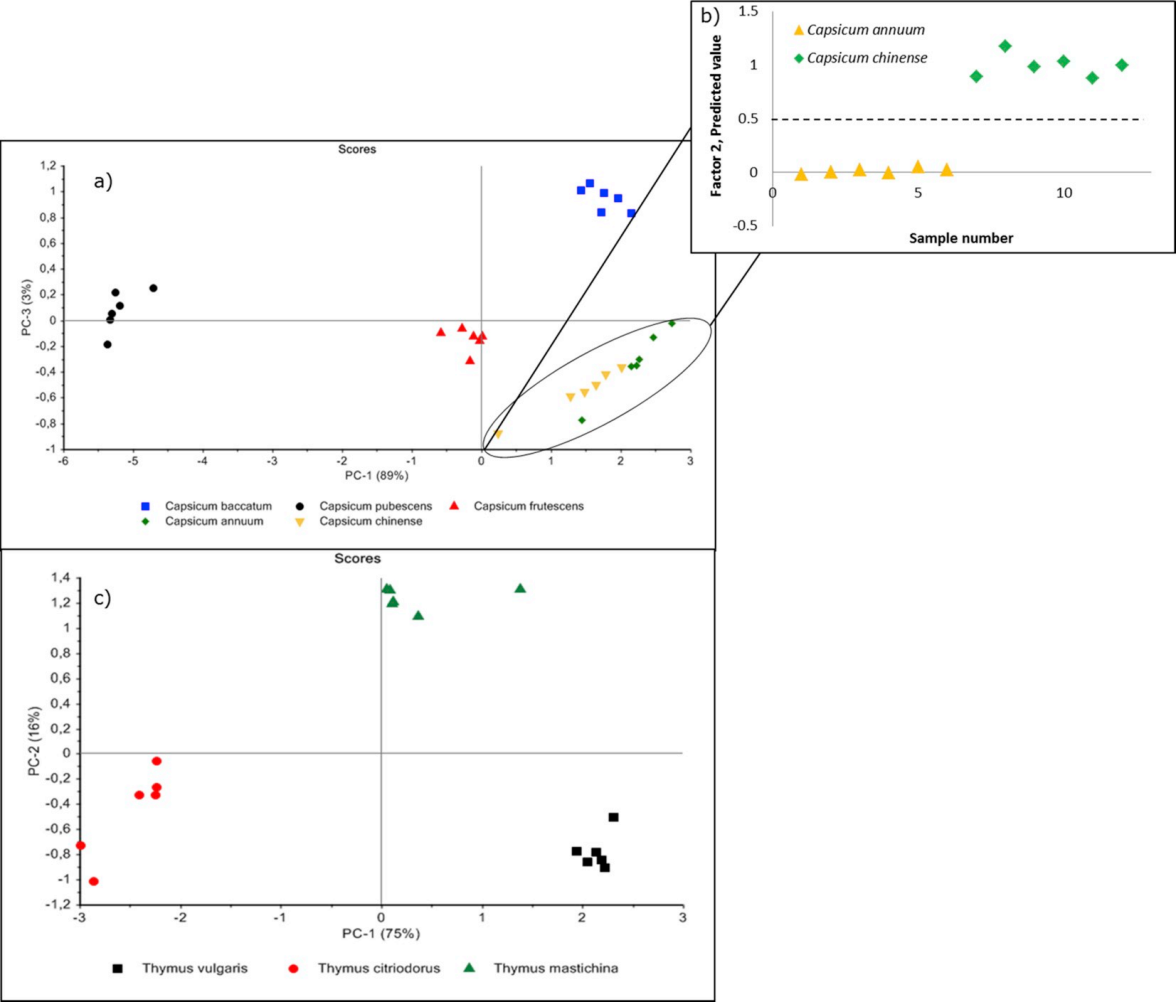
Panel a) Principal component analysis of five *Capsicum* species with 92% of the total variance explained by PC-1 and PC-3. Panel b) Sample grouping of the final PLS-DA with the species *C. annuum* and *C. chinense*. Panel c) A PCA of three *Thymus* species with 91% of the total variance explained by PC-1 and PC-2. Each data point represents a triplicate analysis.

In Fig. 6, Panel a, the PCA shows the clustering of the species *C. annuum*, *C. baccatum*, *C. chinense*, C*. frutescens*, and *C. pubescens* with 92% of the total variance explained by principal components PC-1 (89%) and PC-3 (3%). As expected from the melting profiles, the species *C. baccatum*, *C. frutescens*, and *C. pubescens* were well separated whereas the *C. annuum* and *C. chinense* were inter-clustered and a partial least square discriminant analysis (PLS-DA) was performed. Fig. 6, Panel b, successfully shows the ability of PLS-DA to discriminate between the species of *C. annuum* and *C. chinense*. These results within the *Capsicum* genus can be attributed to the genetically close relationship between the species *C. annuum* and *C. chinense*, and their larger phylogenetic distance to *C. baccatum*, C*. frutescens*, and *C. pubescens* (Carrizo García et al., 2016; Walsh & Hoot, 2001).

The minimal differences observed in the melting profiles of the *Thymus* species shown in Fig. 5, Panel d, prompted the need to apply PCA on the high-resolution melting profiles. In Fig. 6, Panel c, the PCA clearly discriminated the three species of *T. citriodorus*, *T. mastichina*, and *T. vulgaris* with 91% of the total variance explained by PC-1 (75%) and PC-2 (16%).

### 3.6 Discrimination of sub-species

**Fig. 7:**
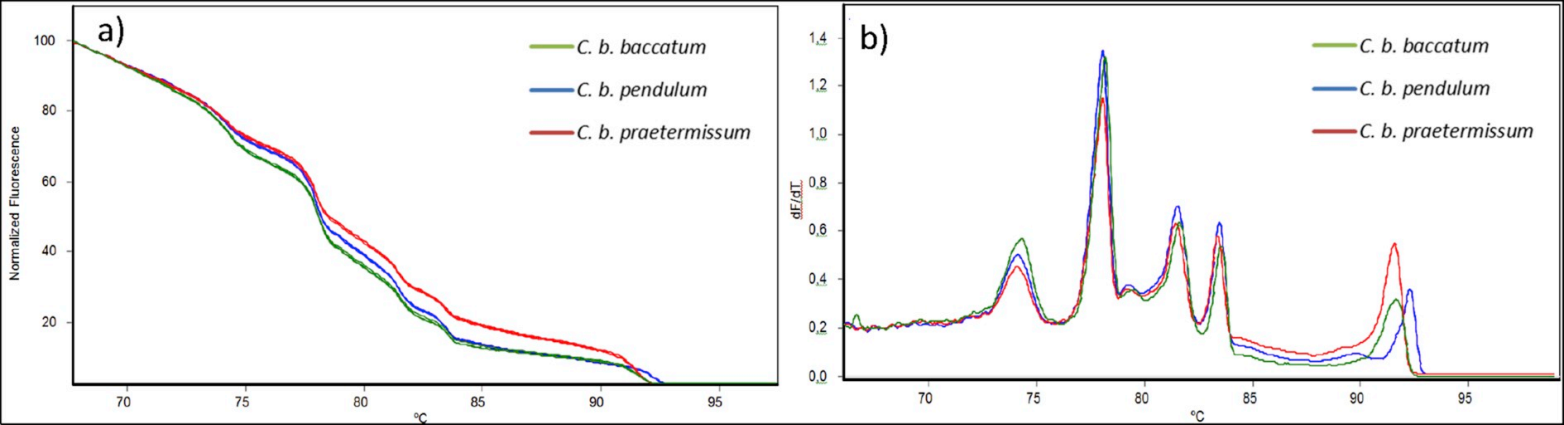
Panel a) Multiplexed high-resolution melting curves of three *Capsicum baccatum* sub-species in triplicate. Panel b) Multiplexed average melting profiles of a triplicate analysis for each sub-species.

Sub-species from *C. baccatum* were analyzed to investigate the discrimination power upon further decreasing the genetic variation. The high-resolution melting profiles presented in Fig. 7, Panel a, show that the melting transition, particularly around 92 °C varied (±0.2 °C). Not surprisingly, this region corresponds to the nuclear *ITS2* (Supplementary S2), which generally possesses a larger discrimination power at the low taxonomic level when compared to the plastid regions (Hollingsworth et al., 2011). Nonetheless, the sub-species of *C. b. pendulum* could be discriminated from *C. b. baccatum* and *C. b. praetermissum,* as the melting transitions at 92.3 °C and 91.6 °C respectively, clearly differed. It was also clear from Fig. 7, Panel b, that *C. b. praetermissum* had a larger intensity, at melting transition 91.6 °C compared to *C. b. baccatum*, and it was therefore not surprising that the PCA discriminated the three sub-species; 98% of the total variance explained by PC-1 (72%) and PC-2 (26%) (data are not shown). However, the different intensities observed at 91.6 °C for the *C. b. baccatum* and *C. b. praetermissum* were probably the result of different copy numbers of the *ITS2* barcode region. It is, therefore, not certain that discrimination based on intensities of the *ITS2* barcode transition would always work, especially as the copy number of the ribosomal genes varies not only between species but also within species; almost a 100-fold difference in copy number was observed in *Vicia faba* (fava bean) (Rogers & Bendich, 1987). More experiments are required to clarify the impact of the intensities on the discrimination power.

### 3.7 Discrimination of cultivars

**Fig. 8:**
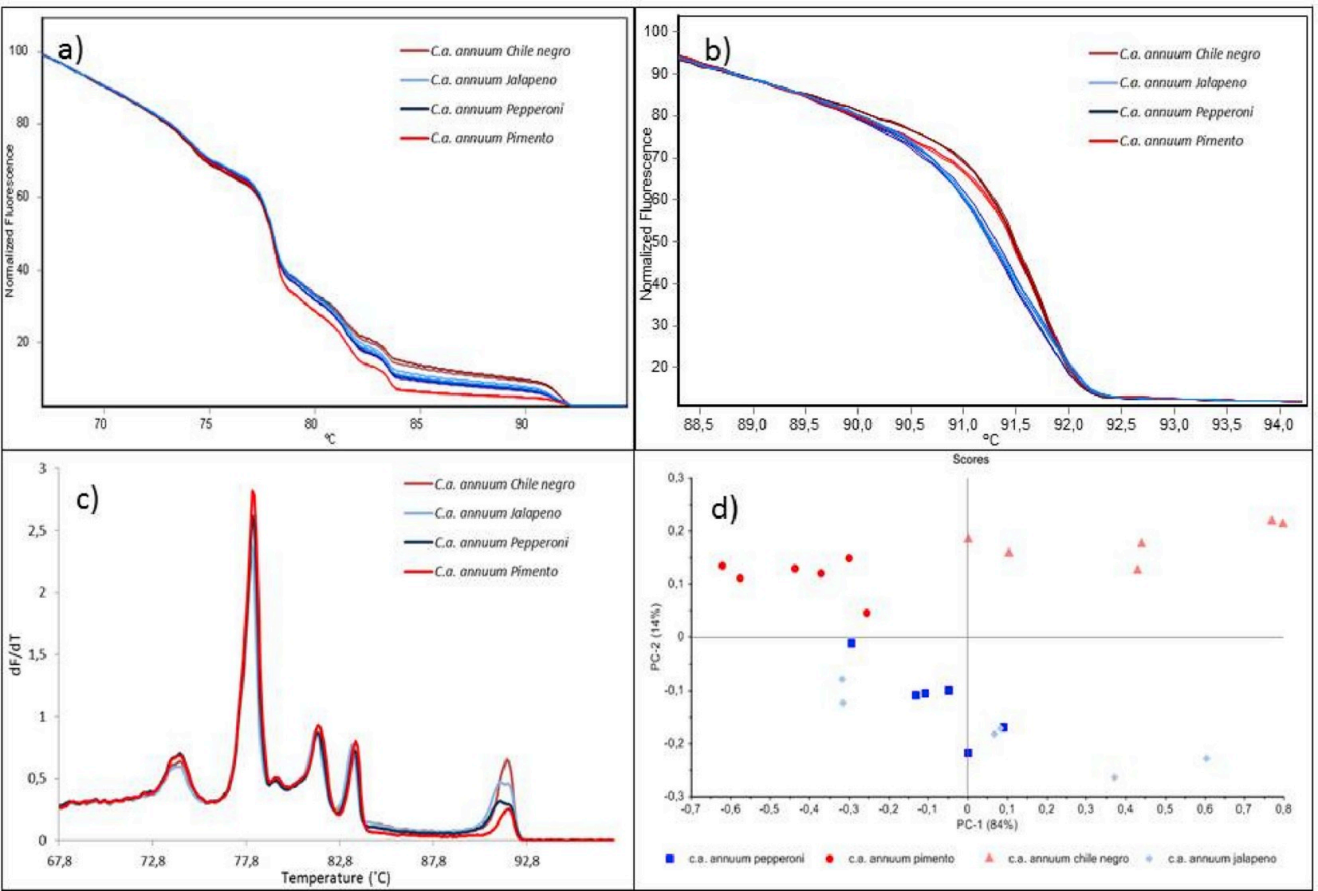
Panel a) Multiplexed high-resolution melting curves representing four cultivars of *Capsicum annuum annuum* in a triplicate analysis. Panel b) The multiplexed high-resolution melting curves, normalized around the 92 °C melting transition. Panel c) Multiplexed average melting profiles of a triplicate analysis. Panel d) PCA of the four cultivars with 98% of the total variance explained by PC-1 and PC-2. Each data point represents a triplicate analysis.

Different cultivars from the *C. annuum annuum* sub-species were analyzed to challenge the approach to its extreme. Again, it was clear that melting, especially of the *ITS2* amplicon (92 °C) offered some discrimination power, Fig. 8, Panel a, shows the normalized high resolution melting in triplicate. To highlight the difference around the 92 °C melting transition, we expanded this region and normalized just before and after the transition, Fig. 8, Panel b. Fig. 8, Panel c, shows the normal melting curves. The PCA in Fig. 8, Panel d, had 98% of the total variance explained by PC-1 (84%) and PC-2 (14%), and showed the approach's inability to distinguish the four cultivars; nonetheless, the Jalapeno and Pepperoni (below the horizontal zero line) seem to behave differently from the Chile Negro and Pimento.

### 3.8 Repeatability, reproducibility, and intra-population variability

In investigating how different DNA extractions and different analytical series affected the melting profile, three experiments were performed to determine repeatability and reproducibility on *T. vulgaris*. Experiment 1: repeatability was investigated through the analysis of nine DNA extractions performed on the same day with the same equipment, personnel, kit, and analyzed in one analytical series. Experiment 2: reproducibility was investigated through the analysis of six DNA extraction performed on different days with the same equipment, different personnel, different batches of the extraction kit, and analyzed in one analytical series. Experiment 3: The same as Experiment 2 but analyzed in six analytical series on different days. Supplementary S5 presents the maximum, minimum, average, and standard deviation for each of the six melting transitions. As expected, the average temperature of the six melting transitions is similar in Experiment 1–3, with a maximum difference of 0.03 °C. For comparison, the tube-to-tube variation specified for the Rotor-Gene Q instrument is 0.02 °C. The standard deviation of the melting transitions in Experiments 1 and 2 differ inconclusively with no apparent trend. This means that performing DNA extractions on different days has an insignificant effect on the melting transition temperatures. Standard deviations of the melting transition temperature in Experiment 3 increased in five out of six melting transitions compared to Experiments 1 and 2. This means that the analyses performed in different analytical series on different days have a significant influence on the melting transition temperature's standard deviation.

Besides investigating variations introduced by DNA extractions and different analytical series, intra-population variability was examined. DNA from nine different wild plants within the species of *Eupatorium cannabinum* (native to the Botanic Garden Meise, Belgium) was extracted on one day and analyzed in one analytical series. Supplementary S5 presents the maximum, minimum, average, and standard deviation for each of the four melting transitions. The data are not directly comparable to Experiment 1 performed on *T. vulgaris*, but it is clear that the main difference is the melting transition intensities. One explanation for this difference could be plant-specific variations in the copy numbers, which is a well-known phenomenon (Rogers & Bendich, 1987). Another possibility could be the variation in DNA purity across the nine extractions.

## 4 Conclusion and perspectives

The presented multiplex Bar-HRM approach provides a DNA profiling platform for species authentication throughout the plant kingdom. Since the approach targets a broad range of species, an in-depth validation and intraspecific variability of individual species was out of scope; the intention was merely to provide a proof of concept for this cheap and high-throughput authentication approach. Also, it should be stressed that the creation of a library is necessary to identify a truly unknown species, or that authentic material should be used in a comparison to verify whether a claim made on a product is valid.

Complex melting profiles were obtained through a multiplexed PCR and a subsequent high-resolution melting of barcode regions of various sequences and sizes. Universality was demonstrated through amplification of 29 species representing different families within the angiosperms and non-angiosperms. Melting and high-resolution melting within the Lamiaceae and the Solanaceae families (especially the *Capsicum* genus) demonstrated discrimination of species, sub-species, and partly cultivars. Thorough attention must be paid to this comprehensive discrimination, and plant references used for sample comparison must be finely selected and annotated to sub-species and cultivar. Species discrimination is primarily based on the difference in melting transition temperatures, but a variation of the intensity of individual melting transitions could, for some species, provide an extra parameter for discrimination. How the copy numbers are translated into intensities of melting transitions is not well established, and the extent to which it can be used as a discriminative parameter has to be further investigated. The multiplex Bar-HRM approach presented has not only shown a huge potential within plant species authentication, but could equally be developed for authentication of animals, fungi, and bacterial species.
