# Supplementary material for: High-resolution melting of multiple barcode amplicons for plant species authentication
Source: Food Control. 2019 Nov;105:141–50. doi: 10.1016/j.foodcont.2019.05.022 (PMC6686639; doi:10.1016/j.foodcont.2019.05.022)
Supplement: Multimedia component 1 [file mmc1.pdf]

**Supplementary 1.** Plant material collected

**Plant species for evaluation of the simplexed reactions**

| Species | Variety | Accession number | Plant material collected | Institution |
|---|---|---|---|---|
| *Capsicum annuum* | Pepperoni | CAP68 | Seeds | Leibniz Institute of Plant Genetics and Crop Plant Research (IPK) OT Gatersleben, Corrensstrasse 3, 06466 Stadt Seeland, Germany |
| *Thymus vulgaris* | Deutche winter | THY1 | Seeds | |
| *Trifolium alpestre* | | 10001617 | Fresh leaves | Botanic Garden Meise, Belgium |

**Plant species for the universality study including 29 families**

| Plant group | Family | Species | Accession number | Plant material collected | Institution |
|---|---|---|---|---|---|
| Angiosperm | Annonaceae | *Annona reticulata* | 20171417-73 | Fresh leaves | Botanic Garden Meise, Belgium |
| Angiosperm | Lauraceae | *Laurus nobilis* | 20140541-43 | | |
| Angiosperm | Apiaceae | *Daucus carota* | 19773292 | | |
| Angiosperm | Apocynaceae | *Adenium multiflorum* | 19802710 | | |
| Angiosperm | Araceae | *Zantedeschia aethiopica* | 20180130-56 | | |
| Angiosperm | Arecaceae | *Elaeis guineensis* | 19514694 | | |
| Angiosperm | Asteraceae | *Stevia rebaudiana* | 19791678 | | |
| Angiosperm | Begoniaceae | *Begonia cubensis* | 19590216 | | |
| Angiosperm | Brassicaceae | *Brassica oleracea* | no number | | |
| Angiosperm | Caryophyllales A | *Nepenthes ventricosa* | 20130007-82 | | |
| Angiosperm | Crassulaceae | *Crassula ovata* | 19514675 | | |
| Angiosperm | Ericaceae | *Vaccinium uliginosum* | 19781981 | | |
| Angiosperm | Euphorbiaceae | *Euphorbia esula* | 19580397 | | |
| Angiosperm | Fabaceae | *Phaseolus vulgaris* | 19832448 | | |
| Moss | Hypnaceae | *Hypnum cupressiforme* | no number | | |
| Angiosperm | Lamiaceae | *Origanum vulgare* | 19580630 | | |
| Angiosperm | Malvaceae | *Theobroma cacao* | 19494807 | | |
| Liverwort | Marchantiaceae | *Marchantia polymorpha* | no number | | |
| Angiosperm | Melastomataceae | *Melastoma sanguineum* | 20041378-14 | | |
| Angiosperm | Orchidaceae | *Vanilla planifolia* | 19074343 | | |
| Angiosperm | Oxalidaceae/Connaraceae | *Oxalis fontana* | 20081449-24 | | |
| Gymnosperm | Pinaceae | *Pinus cembra* | 10001155 | | |
| Angiosperm | Piperaceae | *Piper nigrum* | 19074190 | | |
| Angiosperm | Poaceae | *Zea mays* | 19852616 | | |
| Fern | Pteridaceae | *Dicksonia antarctica* | 19970018-46 | | |
| Angiosperm | Ranunculaceae | *Thalictrum flavum subsp. glaucum* | 19695407 | | |
| Angiosperm | Rosaceae | *Prunus grayana* | 20101124-08 | | |
| Angiosperm | Sapindaceae | *Litchi chinensis* | 19753020 | | |
| Moss | Selaginellaceae | *Selaginella krausiana* | 20050181-87 | | |
| Angiosperm | Solanaceae | *Lycopersicum esculentum* | 10000988 | | |

**Plant species from different genera within the Lamiaceae and the Solanaceae families**

| Genera within the Lamiaceae family | Species | Accession number | Plant material collected | Institution |
|---|---|---|---|---|
| *Mentha* | *Mentha piperita* | 19630527 | Fresh leaves | Botanic Garden Meise, Belgium |
| *Origanum* | *Origanum vulgare* | 19580630 | Fresh leaves | Botanic Garden Meise, Belgium |
| *Salvia* | *Salvia viridis* | 20081801-85 | Fresh leaves | Botanic Garden Meise, Belgium |
| *Thymus* | *Thymus vulgaris* | 089b | Dried | Council for Agricultural Research and Economics (CREA), Research Centre for Forestry and Wood, Viale S. Margherita 80, 52100 Arezzo, Italy |
| Genera within the Solanaceae family | | | | |
| *Capsicum* | *Capsicum annuum* | CAP 1004 | Seeds | Leibniz Institute of Plant Genetics and Crop Plant Research (IPK) OT Gatersleben, Corrensstrasse 3, 06466 Stadt Seeland, Germany |
| *Cyphomandra* | *Cyphomandra betacea* | 19210311 | Fresh leaves | Botanic Garden Meise, Belgium |
| *Lycopersicum* | *Lycopersicum esculentum* | 10000988 | Fresh leaves | Botanic Garden Meise, Belgium |
| *Physalis* | *Physalis peruviana* | 20041375-11 | Fresh leaves | Botanic Garden Meise, Belgium |

**Plant species from the same genus**

| Genus | Species | Accession number | Plant material collected/ provided | Cultivar | Institution |
|---|---|---|---|---|---|
| *Capsicum* | *Capsicum annuum* L. var *annuum* | CAP 1004 | Seeds | Chile Negro | Leibniz Institute of Plant Genetics and Crop Plant Research (IPK) OT Gatersleben, Corrensstrasse 3, 06466 Stadt Seeland, Germany |
| *Capsicum* | *Capsicum baccatum* L. subsp. *pendulum* (Wild.) Eshbaugh | CAP 267 | Seeds | Aje | |
| *Capsicum* | *Capsicum chinense* Jacq. | CAP 1643 | Seeds | BISBAS | |
| *Capsicum* | *Capsicum frutescens* L. | CAP 763 | Seeds | Ají calilla | |
| *Capsicum* | *Capsicum pubescens* Ruiz & Pav | CAP 357 | Seeds | | |
| *Thymus* | *Thymus citriodorus* | 09A8900018 | Dried | Aureus | Crop Research Institute, Prague 6-Ruzyne, Drnovska 507, Czech Republic |
| *Thymus* | *Thymus mastichina* | NC078585 | Live plants | Mejorana | Agrifood Research and Technology Centre of Aragón, Forest Resources Avda Montanana 930, 50059, Zaragoza, Spain |
| *Thymus* | *Thymus vulgaris L.* | 089b | Dried | Varico I | Council for Agricultural Research and Economics (CREA), Research Centre for Forestry and Wood, Viale S. Margherita 80, 52100 Arezzo, Italy |

**Plant sub-species**

| Species | Sub-species | Accession number | Plant material provided | Institution |
|---|---|---|---|---|
| *Capsicum baccatu* | *baccatum* | CAP 1473 | Seed | Leibniz Institute of Plant Genetics and Crop Plant Research (IPK) OT Gatersleben, Corrensstrasse 3, 06466 Stadt Seeland, |
| *Capsicum baccatum* | *pendulum* (Wild.) Eshbaugh | CAP 1034 | Seed | |
| *Capsicum baccatum* | *praetermissum* (Heiser & P.G.SM.) Hunz. | CAP 1141 | Seed | |

**Plant cultivars**

| Species | Cultivar | Accession number | Plant material provided | Institution |
|---|---|---|---|---|
| *Capsicum annuum* L. var *annuum* | Chile Negro | CAP 1004 | Seeds | Leibniz Institute of Plant Genetics and Crop Plant Research (IPK) OT Gatersleben, Corrensstrasse 3, |
| *Capsicum annuum* L. var *annuum* | Jalapeno | CAP 1130 | Seeds | |
| *Capsicum annuum* L. var *annuum* | Pepperoni | CAP 68 | Seeds | |
| *Capsicum annuum* L. var *annuum* | Pimento | CAP 124 | Seeds | |

**Plant material for the repeatability, reproducibility and intra-population study**

| Genus | Species | Accession number | Plant material provided | Cultivar | Institution |
|---|---|---|---|---|---|
| *Eupatorium* | *Eupatorium cannabinum* | no number | Fresh leaves | None | Botanic Garden Meise, Belgium |
| *Thymus* | *Thymus vulgaris L.* | 089b | Dried | Varico I | Council for Agricultural Research and Economics (CREA), Research Centre for Forestry and Wood, Viale S. Margherita 80, 52100 Arezzo, Italy |
