# Supplementary material for: High-resolution melting of multiple barcode amplicons for plant species authentication
Source: Food Control. 2019 Nov;105:141–50. doi: 10.1016/j.foodcont.2019.05.022 (PMC6686639; doi:10.1016/j.foodcont.2019.05.022)
Supplement: Multimedia component 2 [file mmc2.pdf]

Supplementary 2. Melting curves from amplification of barcode regions *ITS2*, *rbcL*, *trnL* c, d, *trnL* g, h, and the *psbA-trnH* in *Capsicum annuum* and *Trifolium alpestre*.

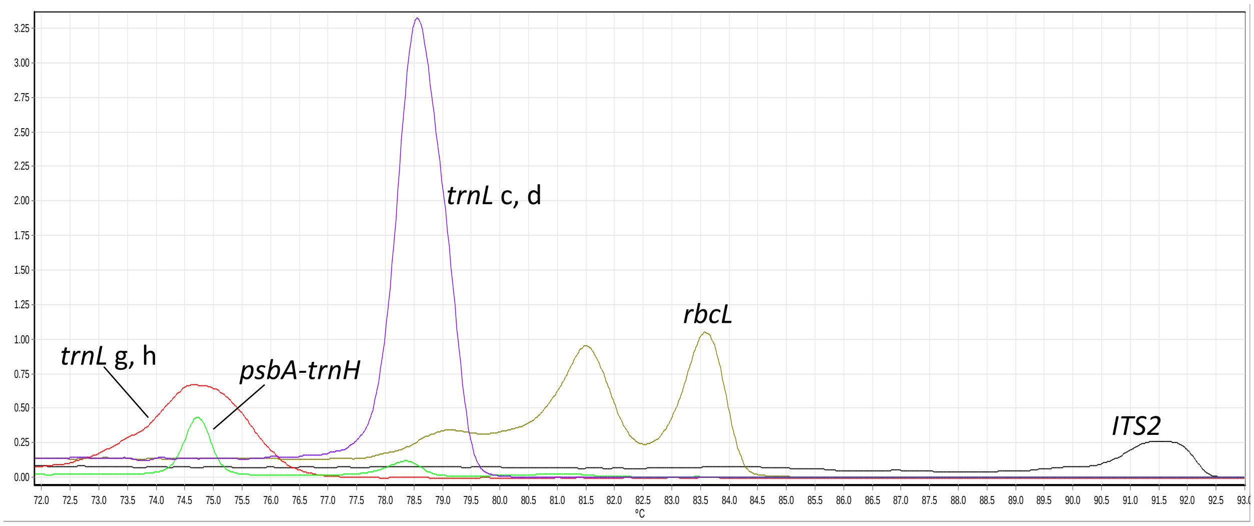

Fig. 1. Simplexed melting *curves from Capsicum annuum*.

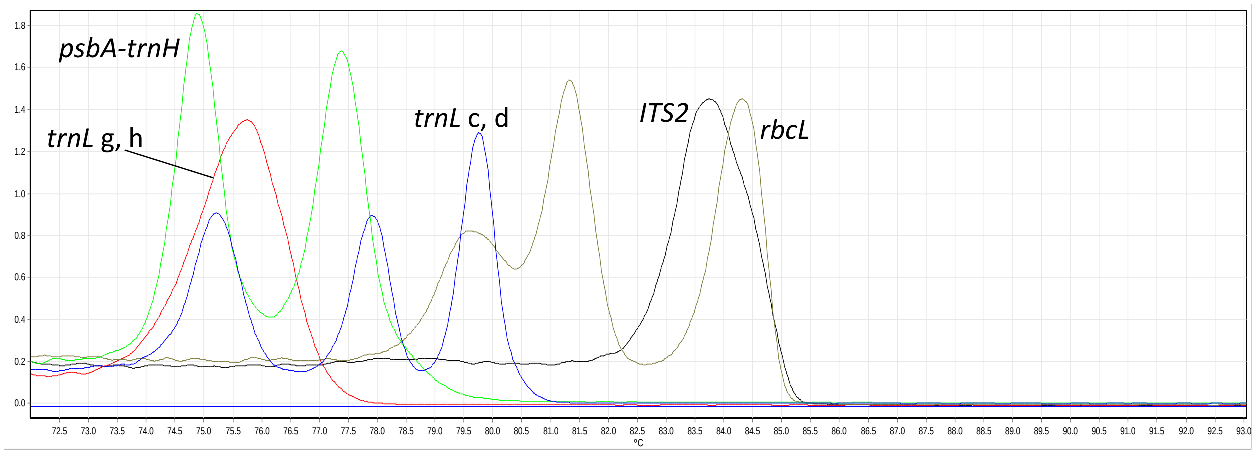

Fig. 2. Simplexed melting curves from *Trifolium alpestre*.
