# Supplementary material for: High-resolution melting of multiple barcode amplicons for plant species authentication
Source: Food Control. 2019 Nov;105:141–50. doi: 10.1016/j.foodcont.2019.05.022 (PMC6686639; doi:10.1016/j.foodcont.2019.05.022)
Supplement: Multimedia component 3 [file mmc3.pdf]

Supplementary 3. uMELT predictions of the number of melting transitions from *Thymus vulgaris* barcode regions. The uMELT software (https://www.dna.utah.edu/umelt/umelt.html) was accessed 12 November 2018. DNA sequences were retrieved 12 November 2018 from the NCBI webpage (https://www.ncbi.nlm.nih.gov/). The sequences used for the uMELT analysis were restricted by the primers used in the article.

Specification for the uMELT software (Dwight, Palais, & Wittwer, 2011) with parameters reflecting the AmpliTaq Gold 360 mastermix (Thermofisher, Foster City CA, USA):

$Mono^{+}$: 16 mM

Free mg^{++}: 3.4 mM

DMSO: 0%

Salt correction: SL and Hicks (2004)

Thermodynamic set:

Unified santaLucia

Temperature range:

Temp: 65 – 101 ^{o}C

Resolution: 0.1 ^{o}C

Table 1. *In silico* uMELT generated melting curves and experimental melting curves obtained from the *ITS2*, *rbcL*, *trnL* c, d and the *trnL* g,h barcode regions restricted by the primers specified in the article

Barcode	Accession	uMELT generated melting curves	Experimental melting curve
ITS2	MF465202.1 and MH688886.1	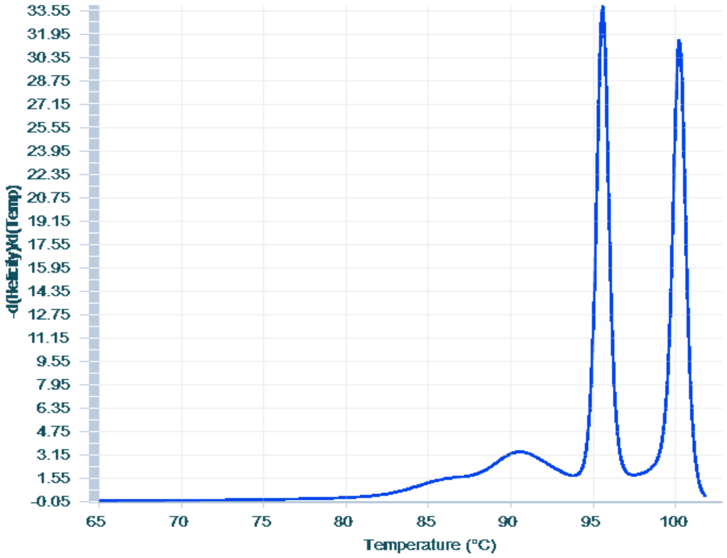	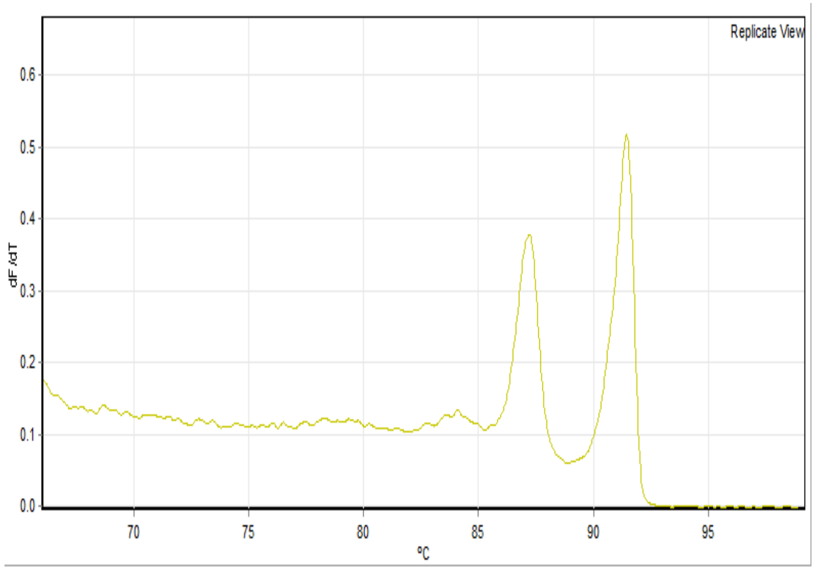

<table>
<tr><th>Barcode</th><th>Accession</th><th>uMELT generated melting curves</th><th>Experimental melting curve</th></tr>
<tr><td>rbcL</td><td>Z37471.1</td><td>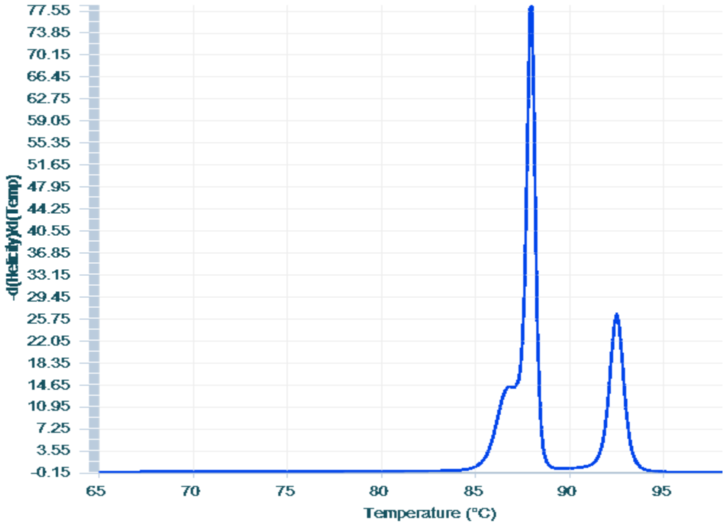
</td><td>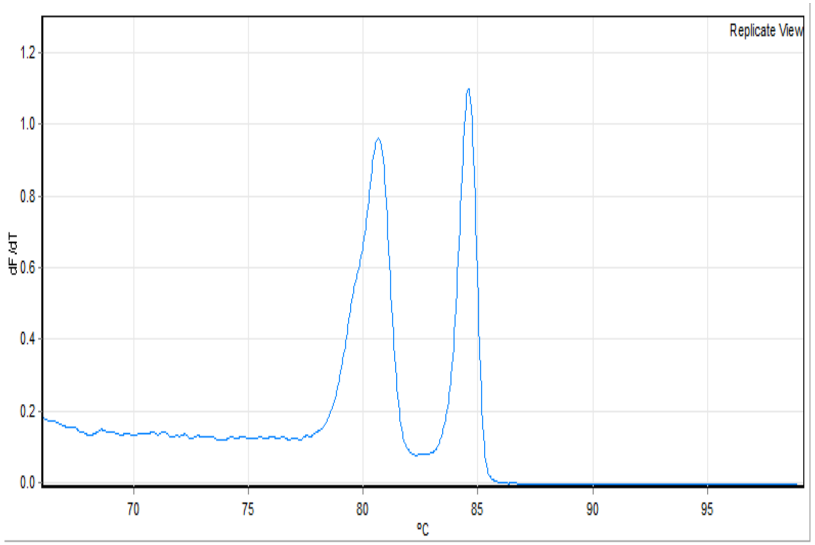
</td></tr>
</table>

Barcode	Accession	uMELT generated melting curves	Experimental melting curve
trnL c, d	EU556530.1 and AY506613.1	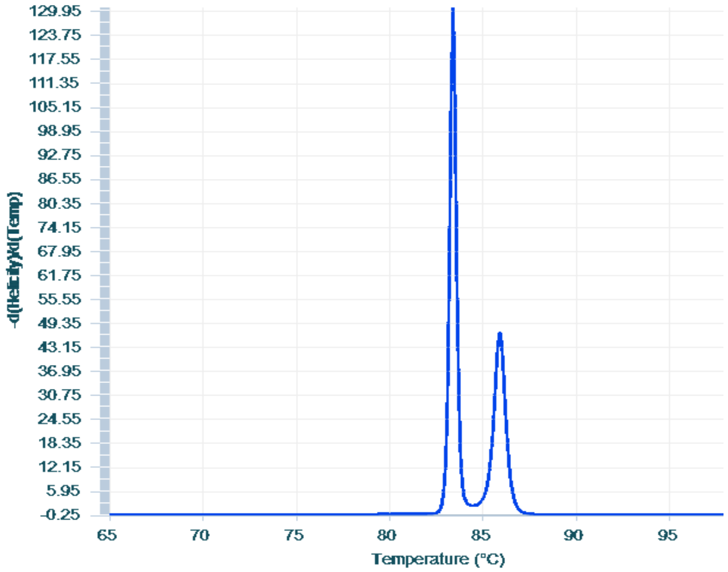	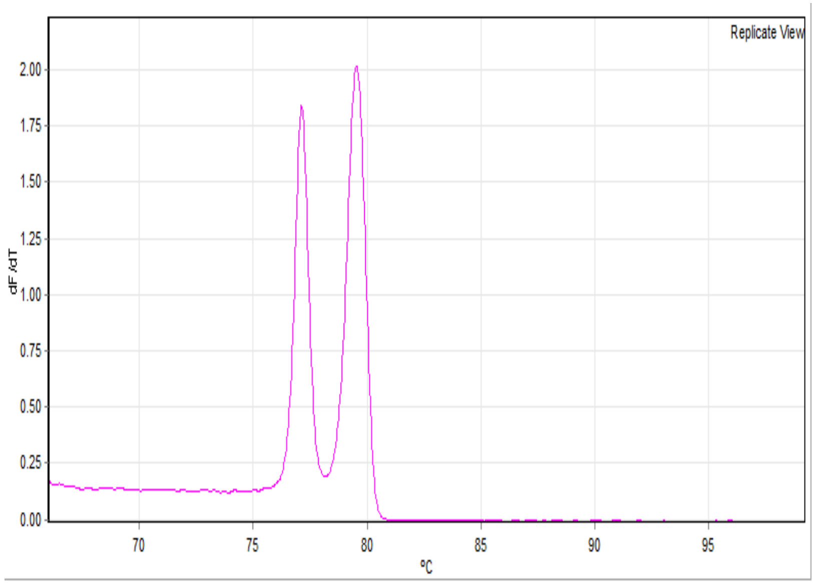

Barcode	Accession	uMELT generated melting curves	Experimental melting curve
trnL g, h	AY506613.1	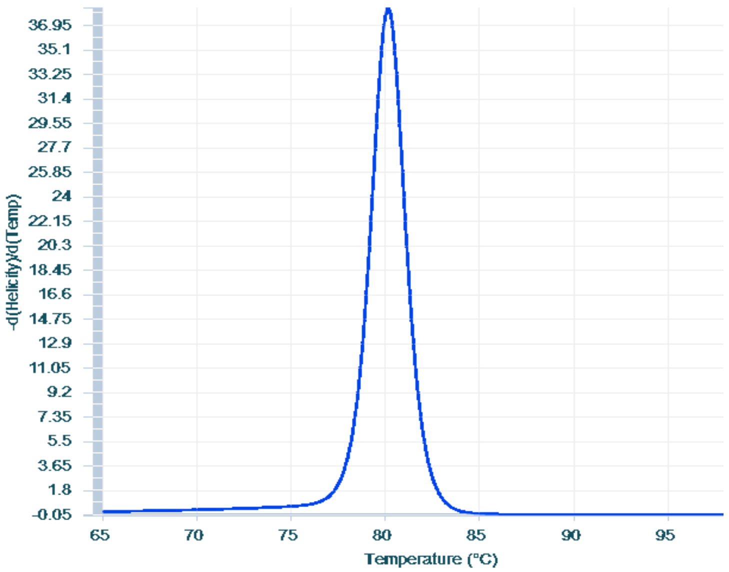	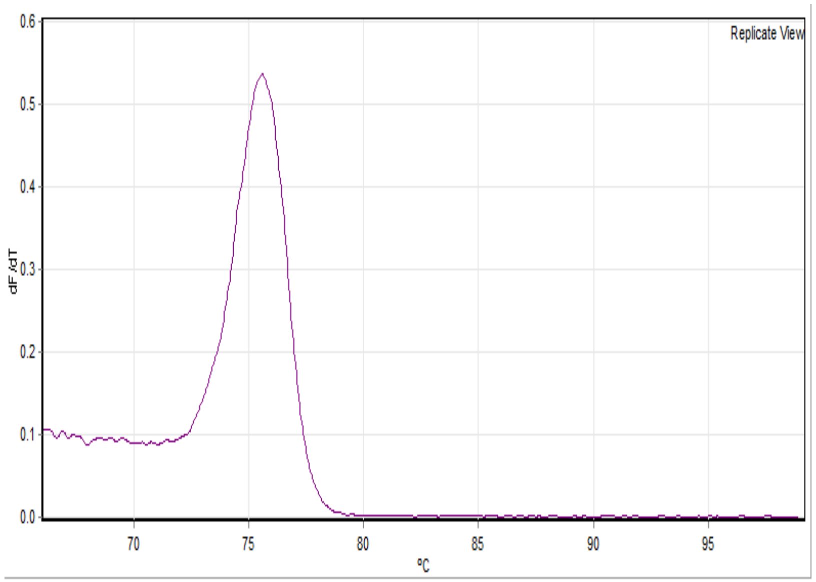

Note:

The differences between theoretical and experimental intensities and absolute melting transition temperatures are obvious. Reasons for these discrepancies are well acknowledged (Dwight, Palais, & Wittwer, 2011).

Reference:

Dwight, Z., Palais, R., & Wittwer, C. T. (2011). uMELT: prediction of high-resolution melting curves and dynamic melting profiles of PCR products in a rich web application. *Bioinformatics, 27*(7), 1019-1020.
