# Supplementary material for: High-resolution melting of multiple barcode amplicons for plant species authentication
Source: Food Control. 2019 Nov;105:141–50. doi: 10.1016/j.foodcont.2019.05.022 (PMC6686639; doi:10.1016/j.foodcont.2019.05.022)
Supplement: Multimedia component 4 [file mmc4.pdf]

Supplementary 4. Multiplexed melting profiles from 29 plant species representing 29 families. Three replicates are shown in each case except for Apiaceae where one replicate was missed in the pipetting.

Family **Species**

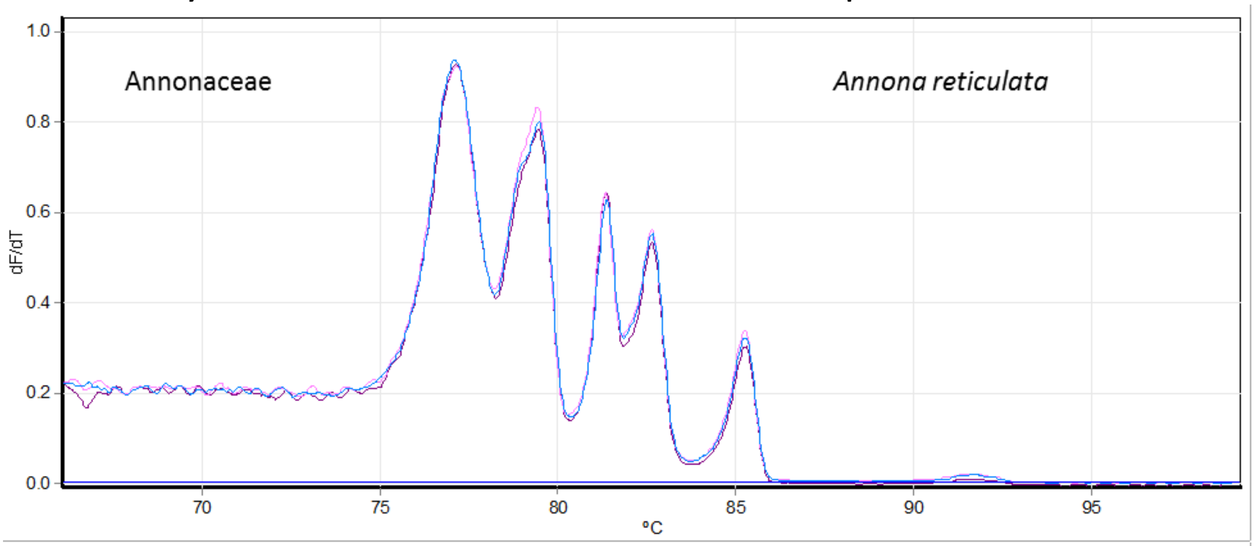

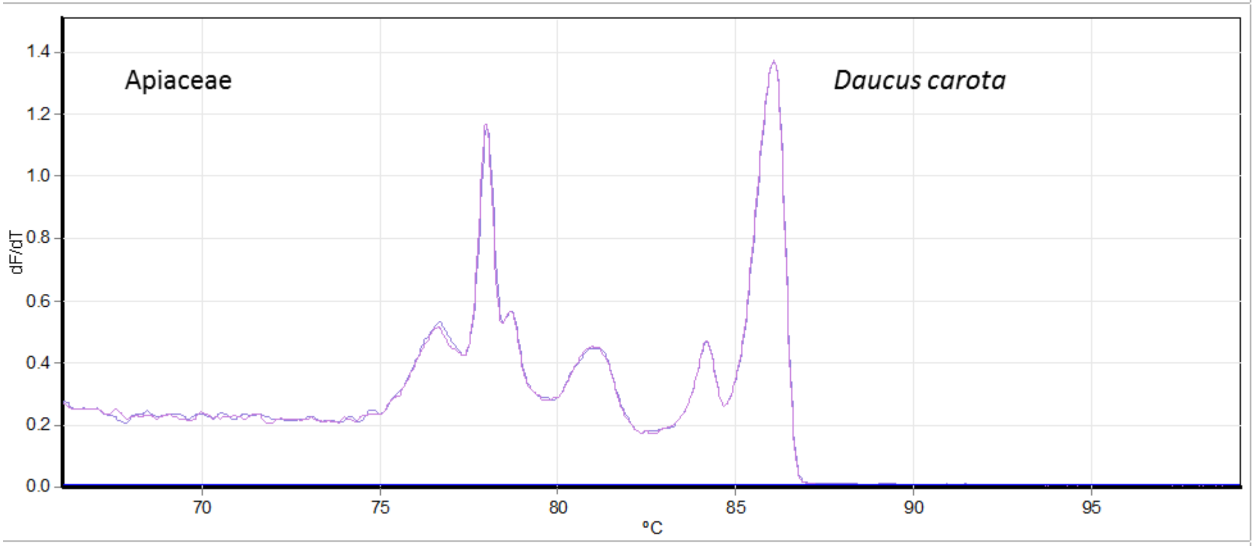

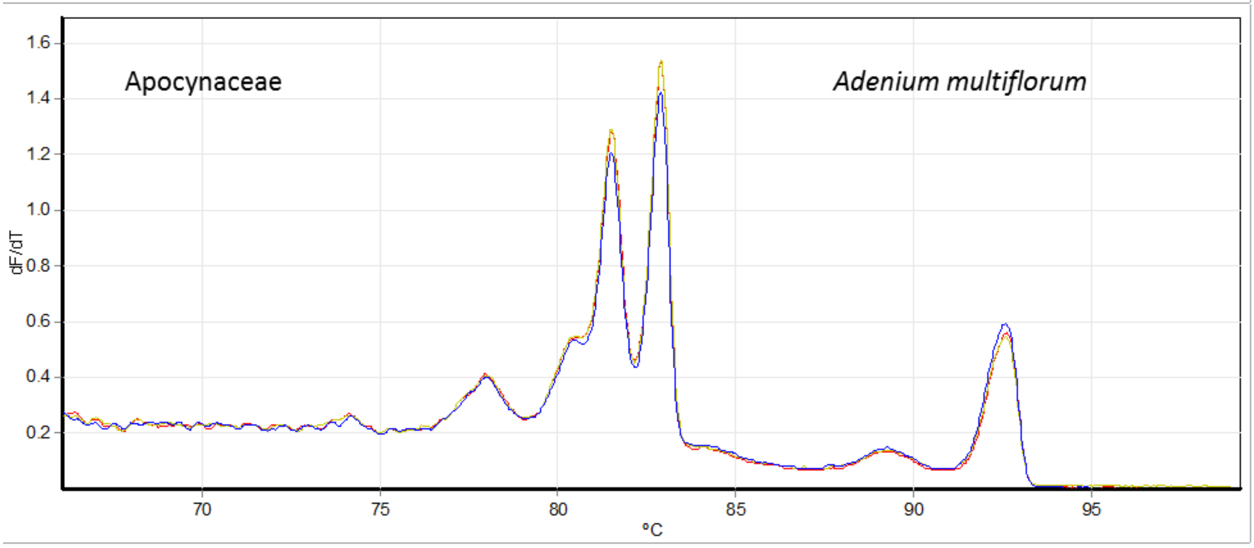

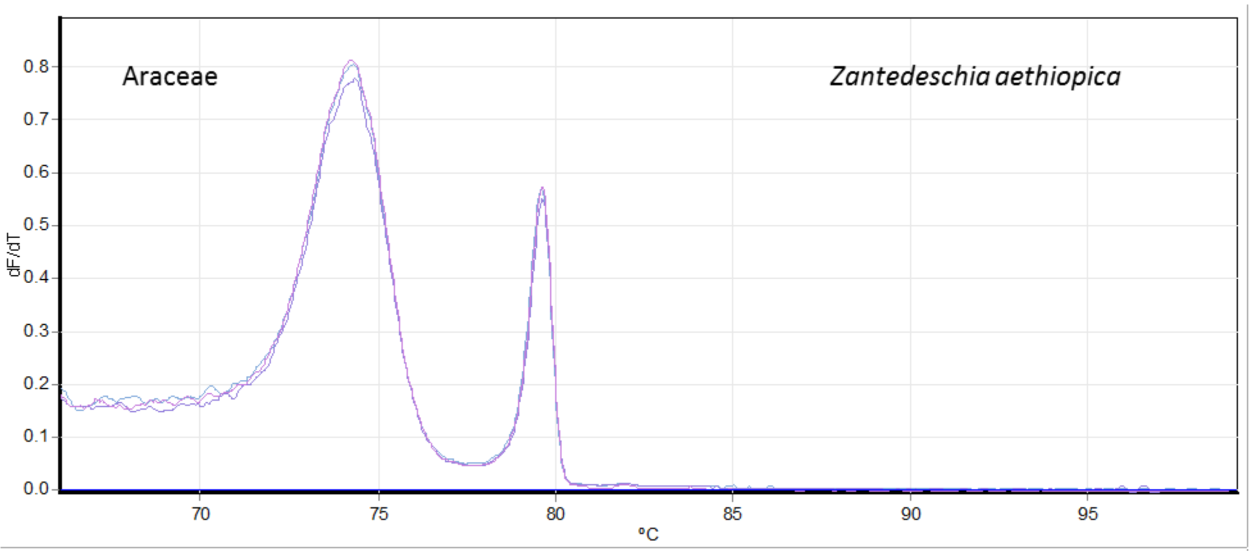

Araceae
Zantedeschia aethiopica
dF/dT
°C

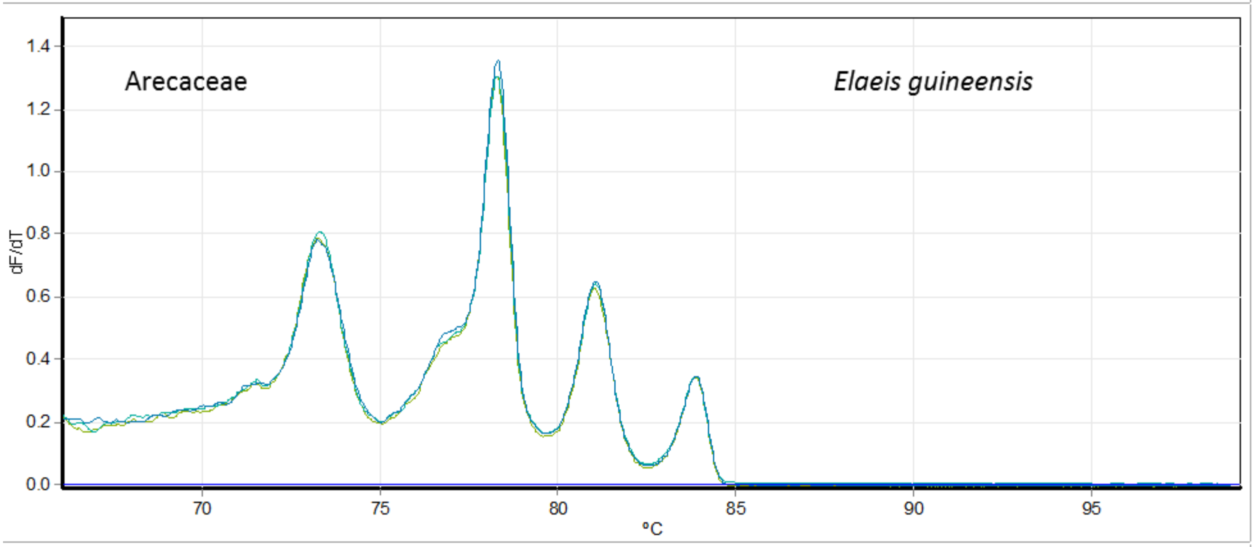

Arecaceae
Elaeis guineensis
dF/dT
°C

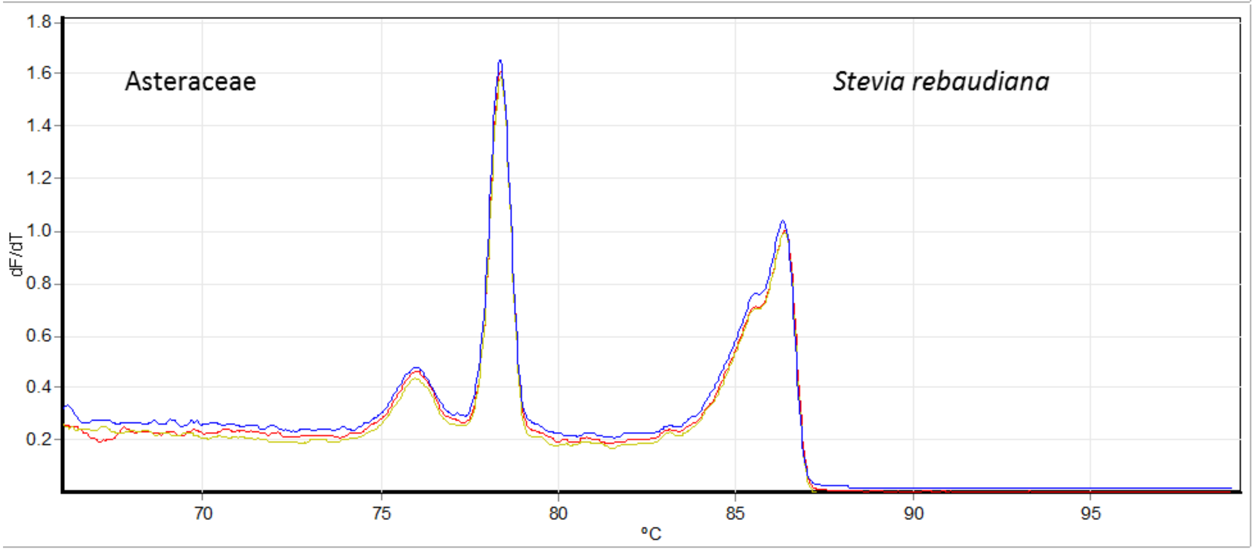

Asteraceae
Stevia rebaudiana
dF/dT
°C

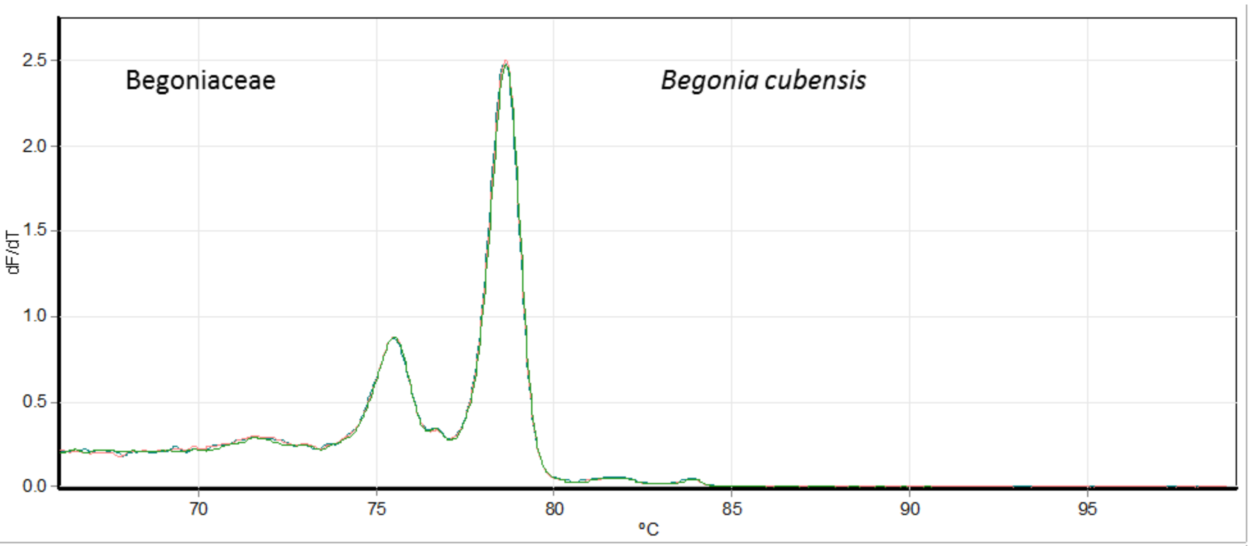
Begoniaceae
Begonia cubensis
dF/dT
°C
2.5
2.0
1.5
1.0
0.5
0.0
70
75
80
85
90
95

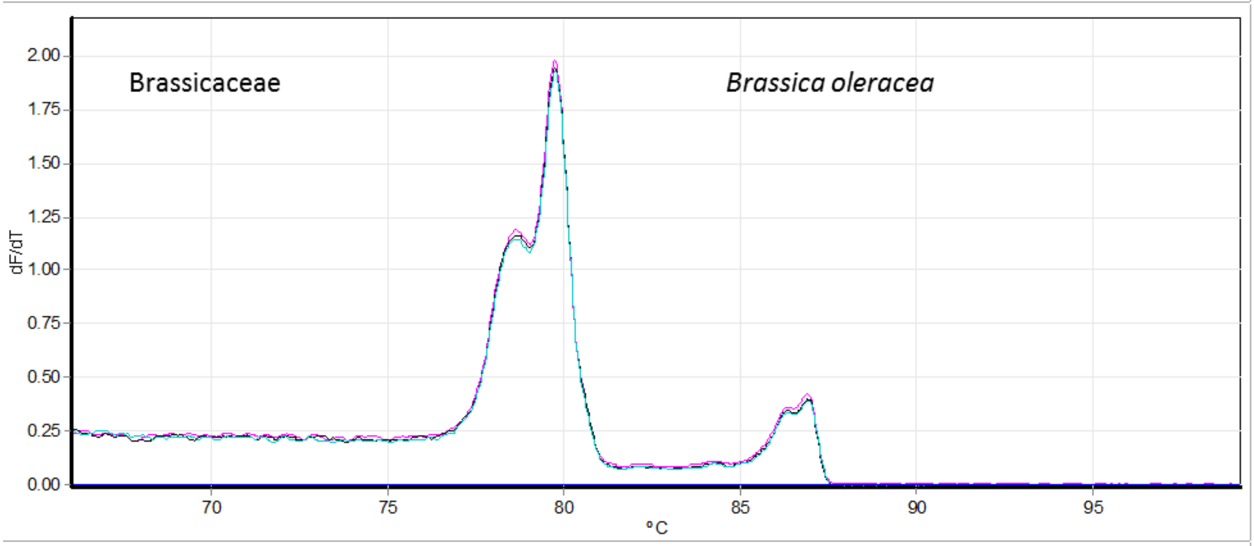
Brassicaceae
Brassica oleracea
dF/dT
°C
2.00
1.75
1.50
1.25
1.00
0.75
0.50
0.25
0.00
70
75
80
85
90
95

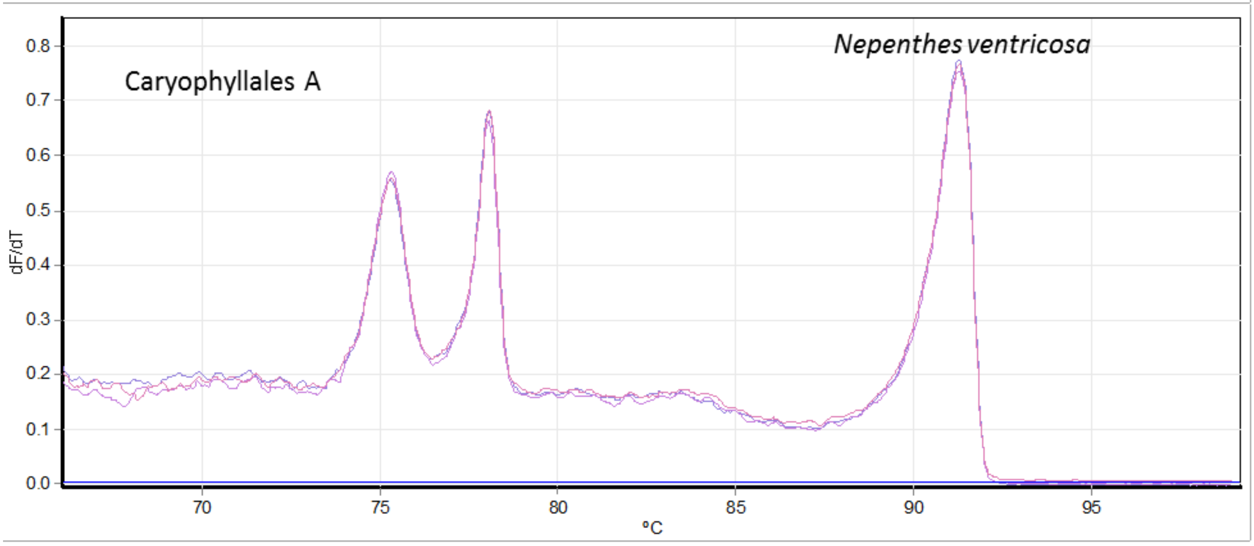
Caryophyllales A
Nepenthes ventricosa
dF/dT
°C
0.8
0.7
0.6
0.5
0.4
0.3
0.2
0.1
0.0
70
75
80
85
90
95

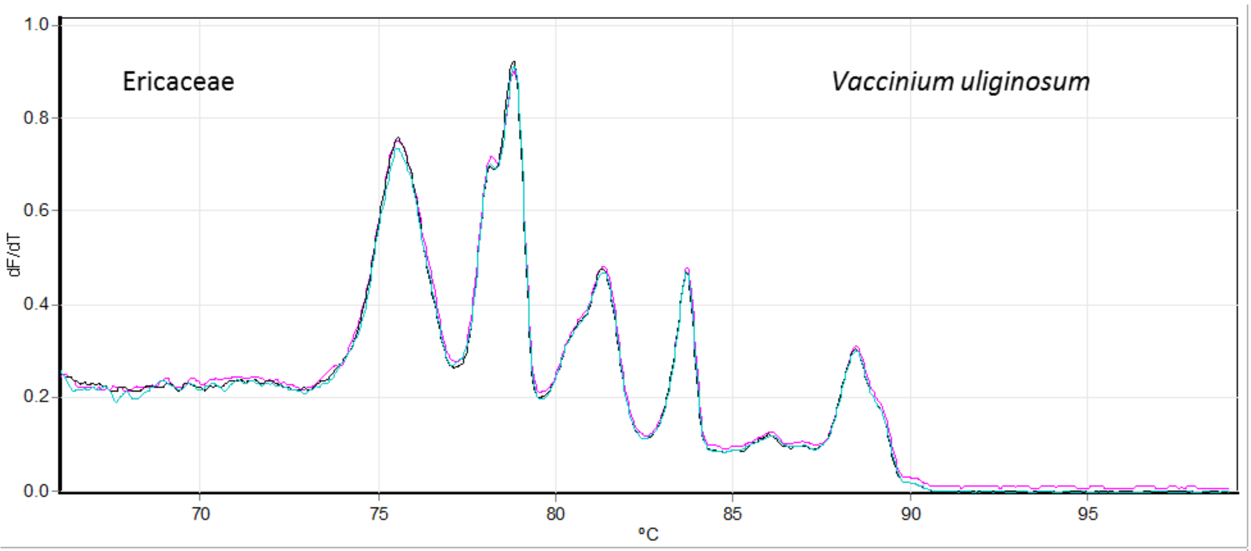
Ericaceae
Vaccinium uliginosum
dF/dT
1.0
0.8
0.6
0.4
0.2
0.0
70
75
80
85
90
95
°C

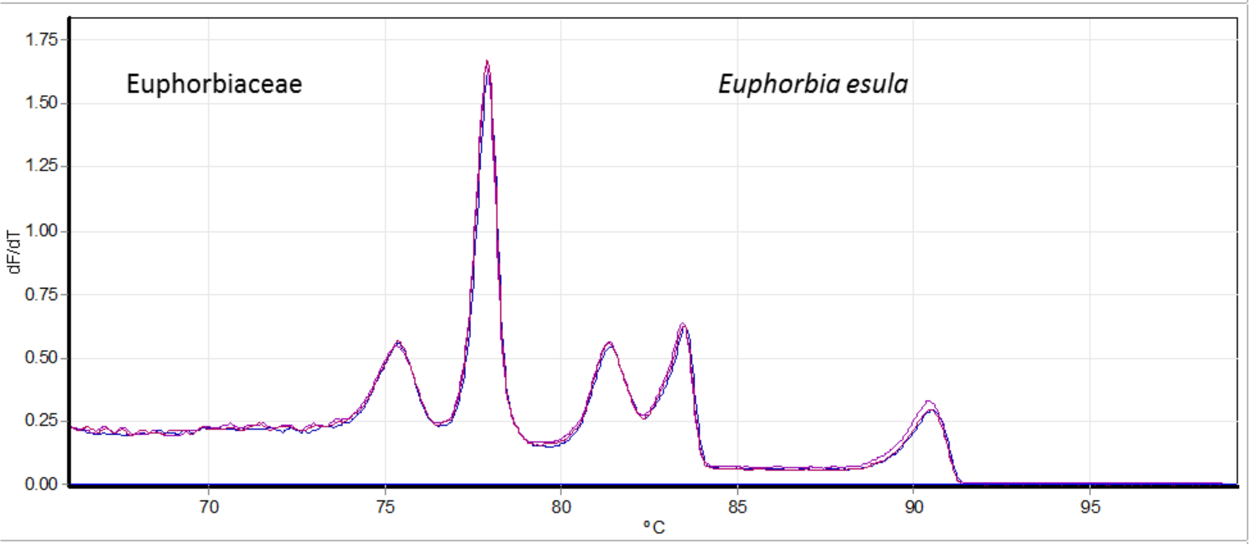
Euphorbiaceae
Euphorbia esula
dF/dT
1.75
1.50
1.25
1.00
0.75
0.50
0.25
0.00
70
75
80
85
90
95
°C

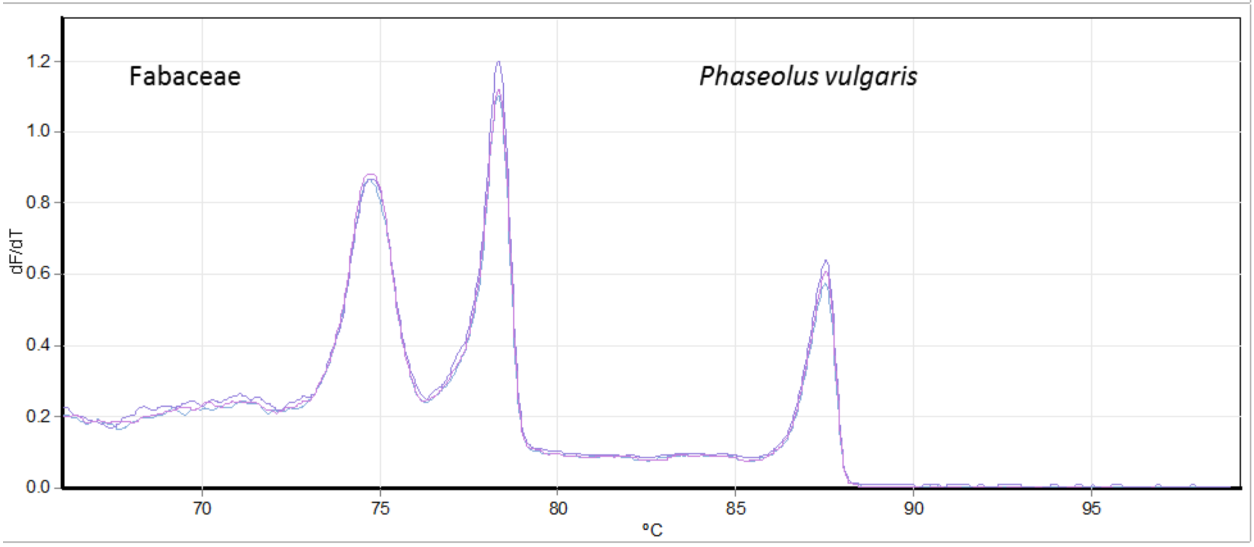
Fabaceae
Phaseolus vulgaris
dF/dT
1.2
1.0
0.8
0.6
0.4
0.2
0.0
70
75
80
85
90
95
°C

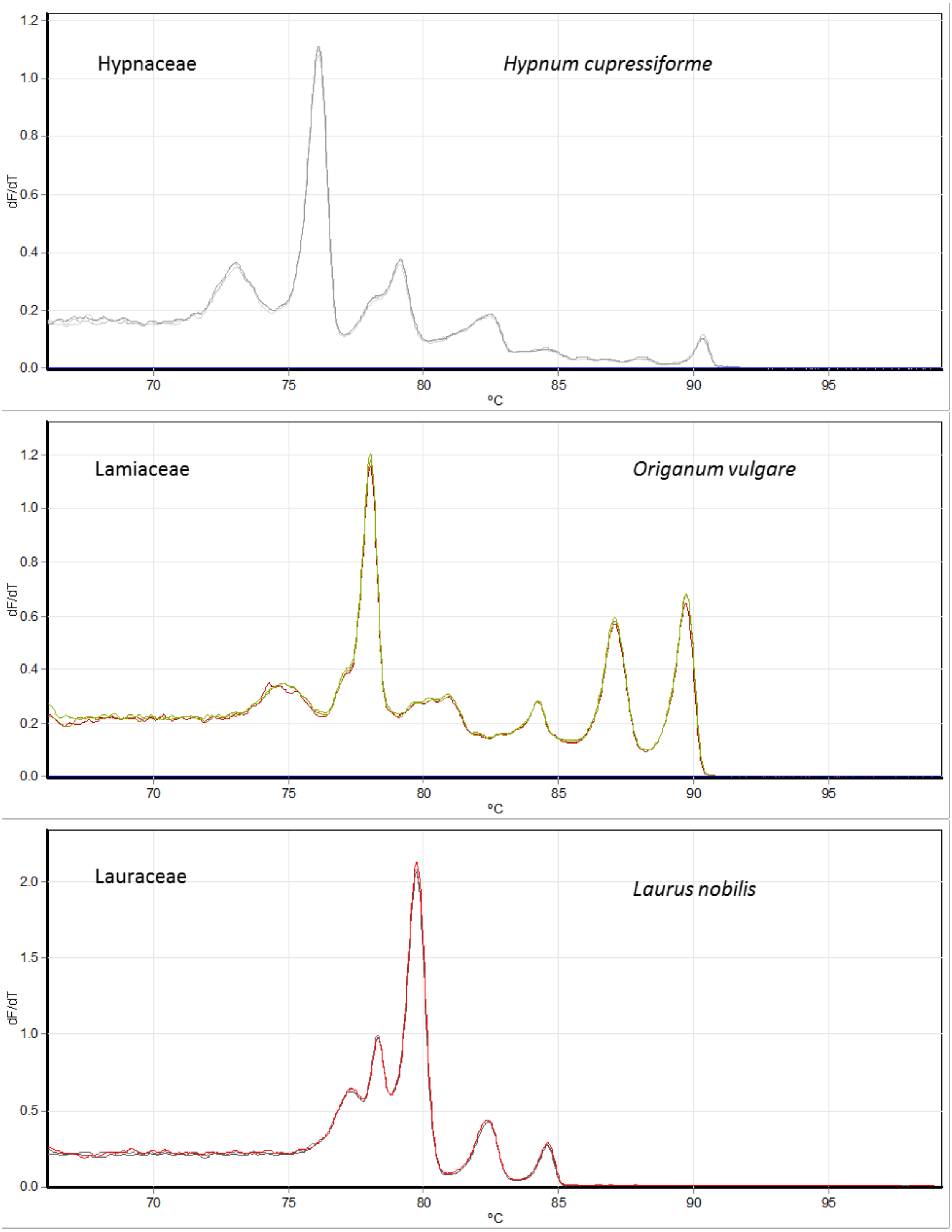
Hypnaceae
Hypnum cupressiforme
dF/dT
°C
Lamiaceae
Origanum vulgare
dF/dT
°C
Lauraceae
Laurus nobilis
dF/dT
°C

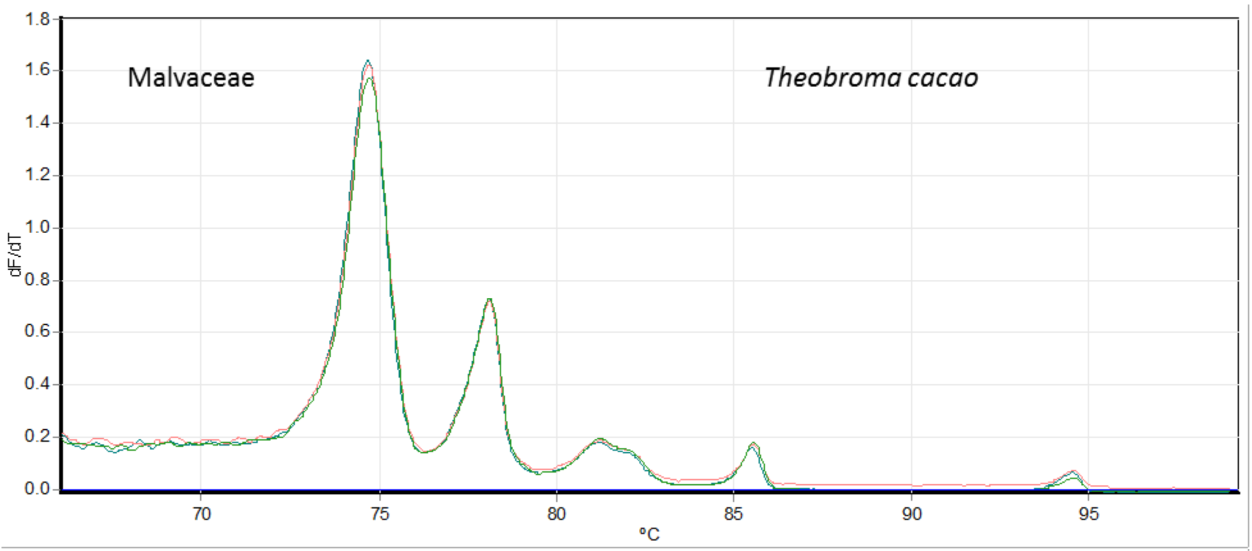
Malvaceae
Theobroma cacao
dF/dT
1.8
1.6
1.4
1.2
1.0
0.8
0.6
0.4
0.2
0.0
70
75
80
85
90
95
°C

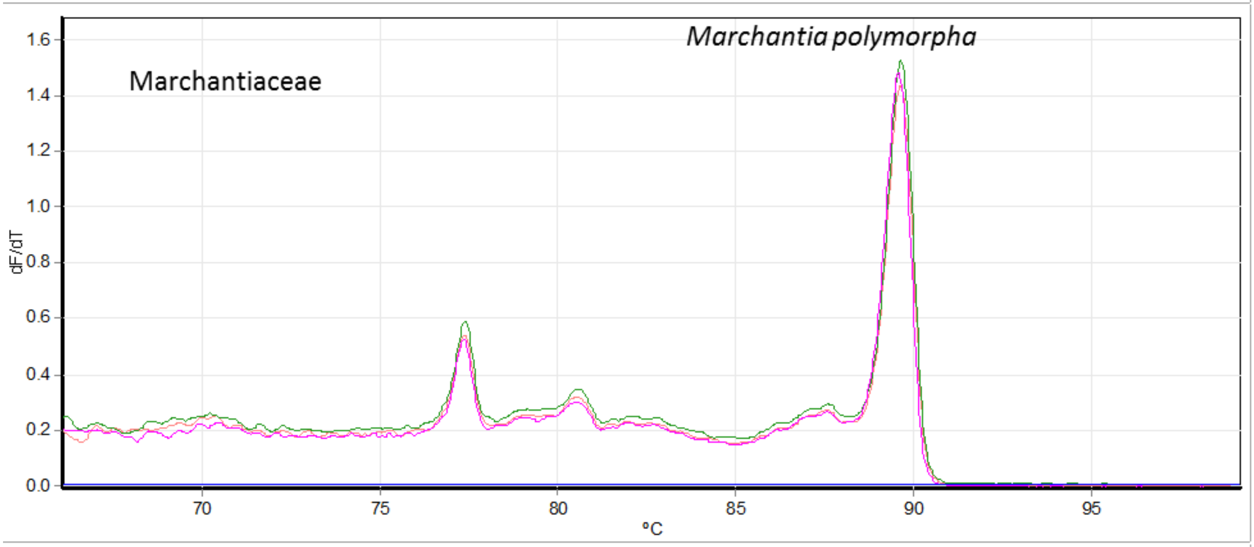
Marchantiaceae
Marchantia polymorpha
dF/dT
1.6
1.4
1.2
1.0
0.8
0.6
0.4
0.2
0.0
70
75
80
85
90
95
°C

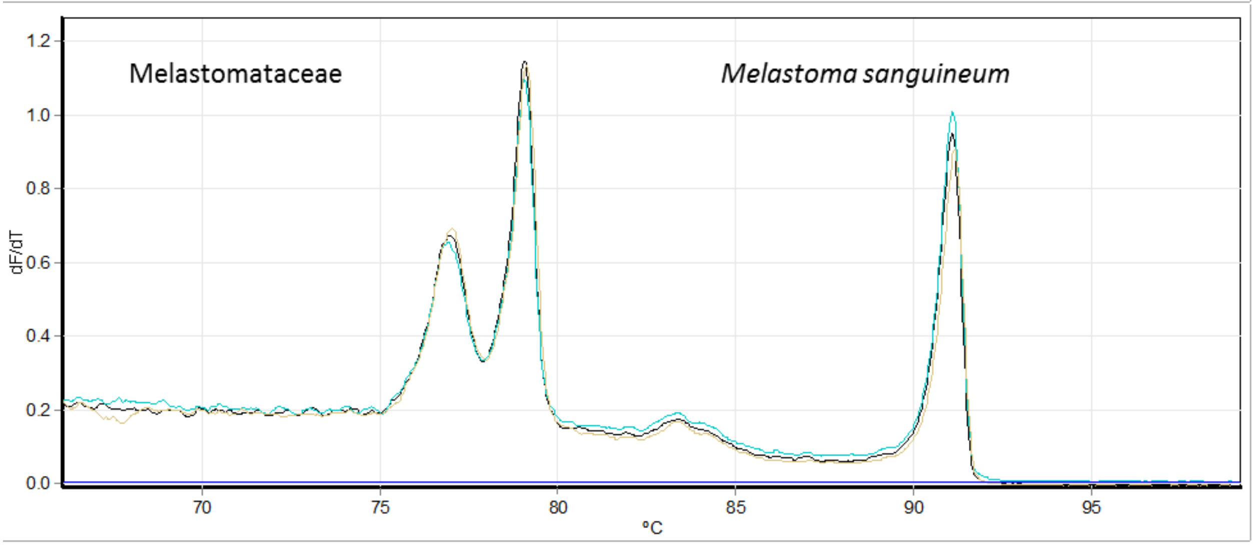
Melastomataceae
Melastoma sanguineum
dF/dT
1.2
1.0
0.8
0.6
0.4
0.2
0.0
70
75
80
85
90
95
°C

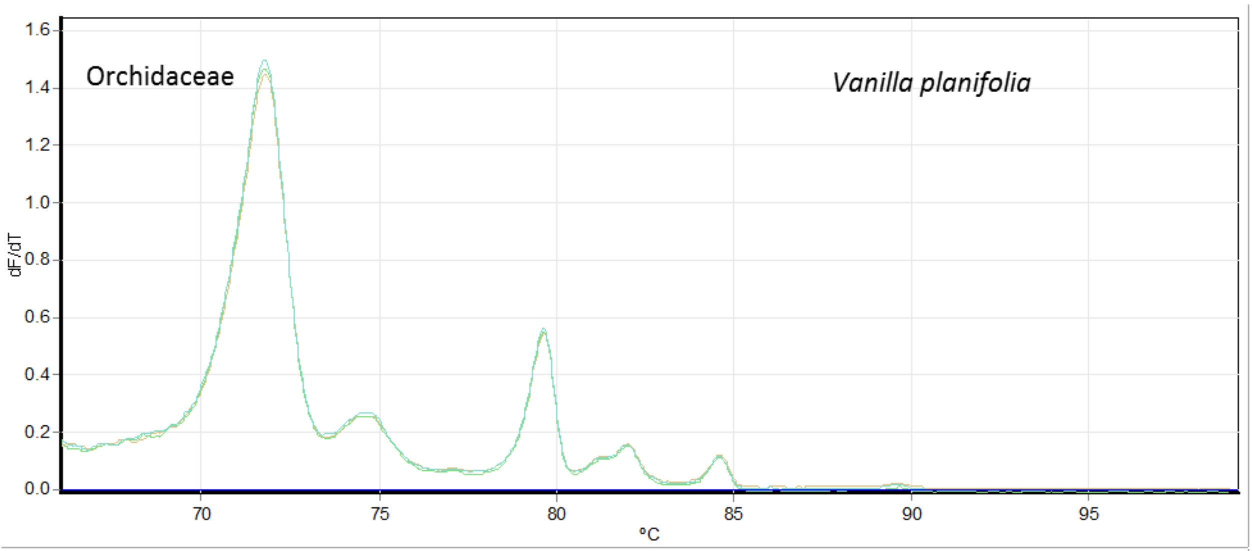
Orchidaceae
Vanilla planifolia
dF/dT
1.6
1.4
1.2
1.0
0.8
0.6
0.4
0.2
0.0
70
75
80
85
90
95
°C

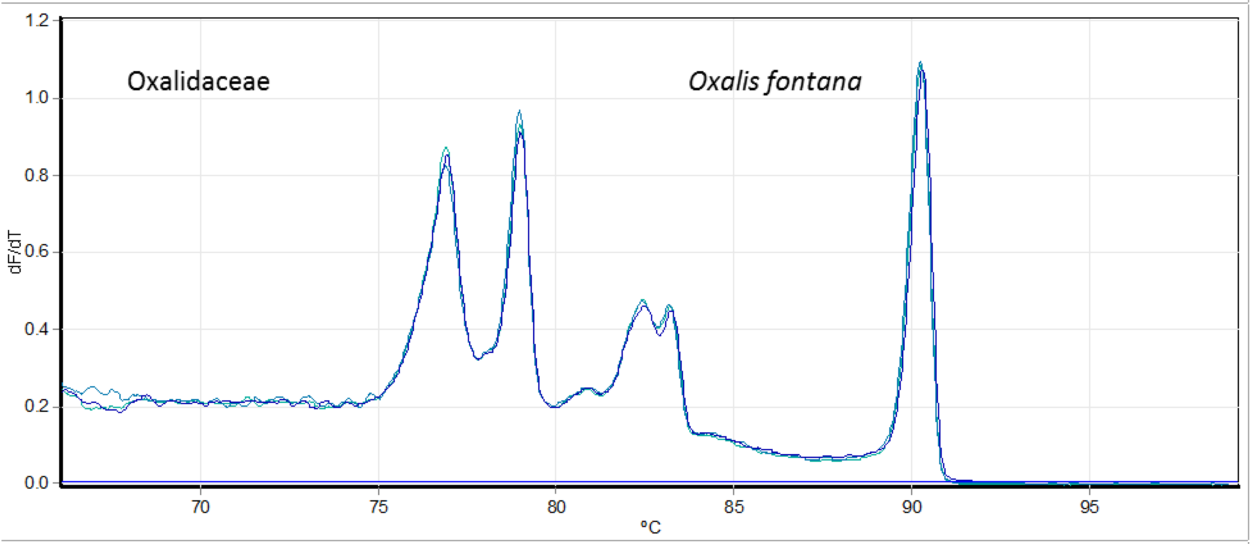
Oxalidaceae
Oxalis fontana
dF/dT
1.2
1.0
0.8
0.6
0.4
0.2
0.0
70
75
80
85
90
95
°C

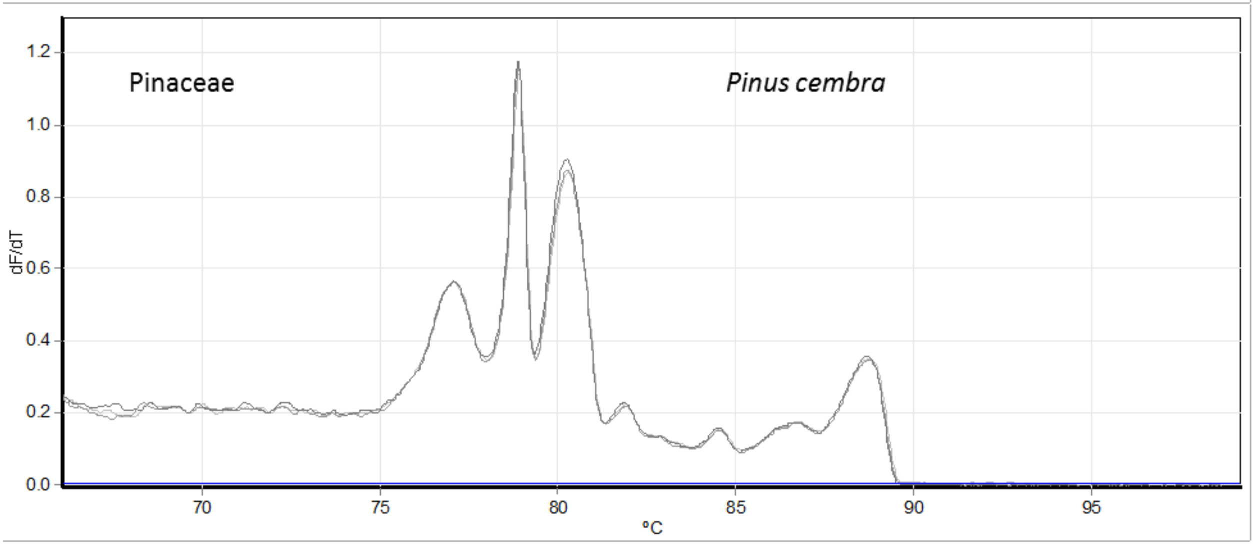
Pinaceae
Pinus cembra
dF/dT
1.2
1.0
0.8
0.6
0.4
0.2
0.0
70
75
80
85
90
95
°C

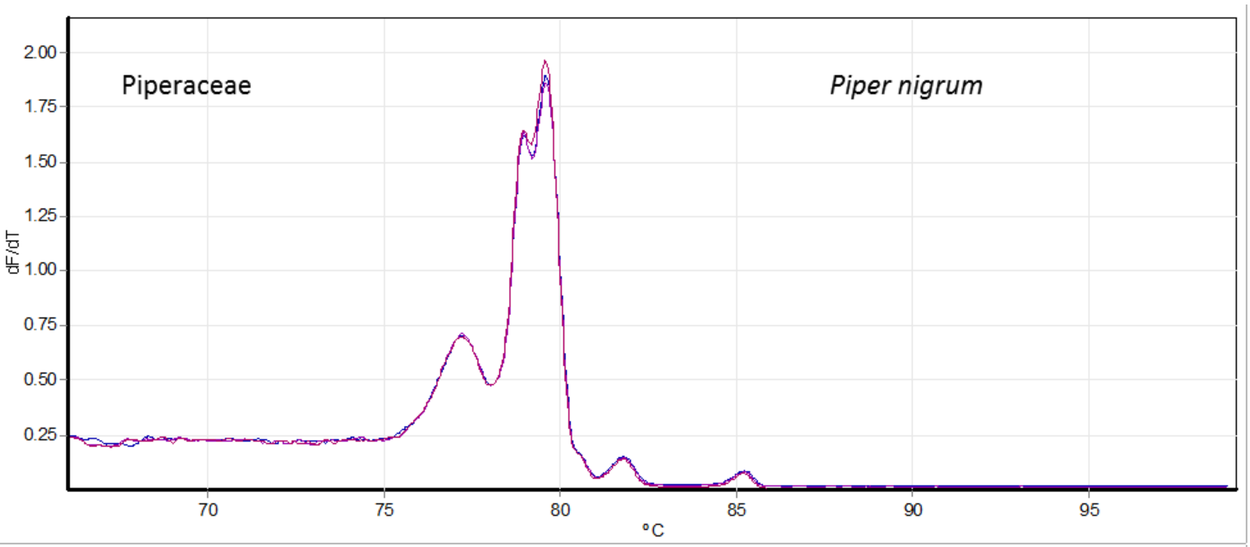
Piperaceae
Piper nigrum
dF/dT
2.00
1.75
1.50
1.25
1.00
0.75
0.50
0.25
70
75
80
85
90
95
°C

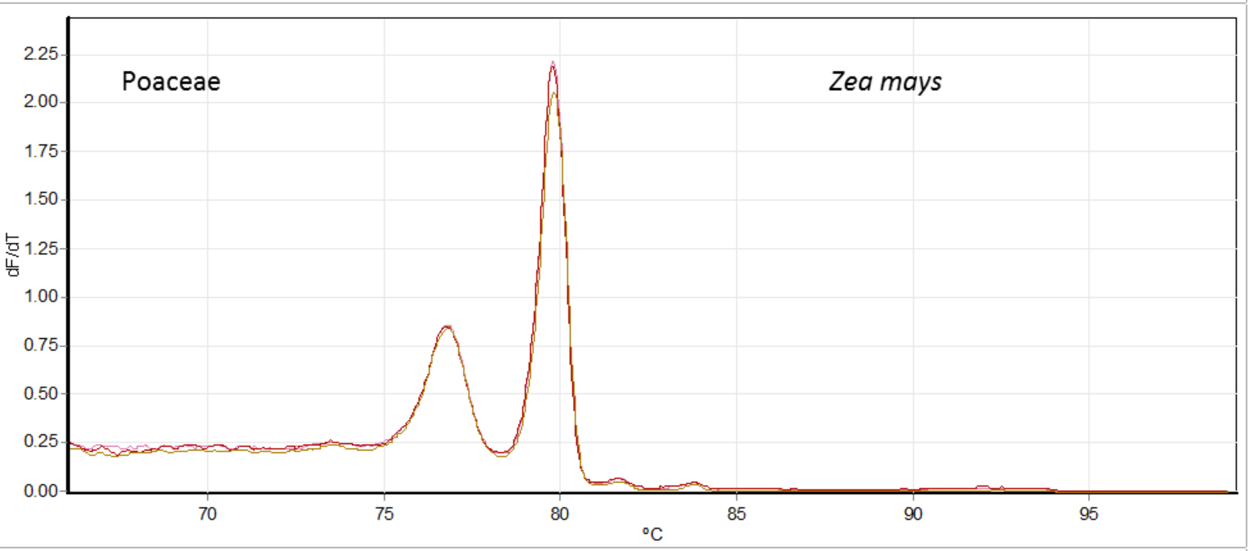
Poaceae
Zea mays
dF/dT
2.25
2.00
1.75
1.50
1.25
1.00
0.75
0.50
0.25
0.00
70
75
80
85
90
95
°C

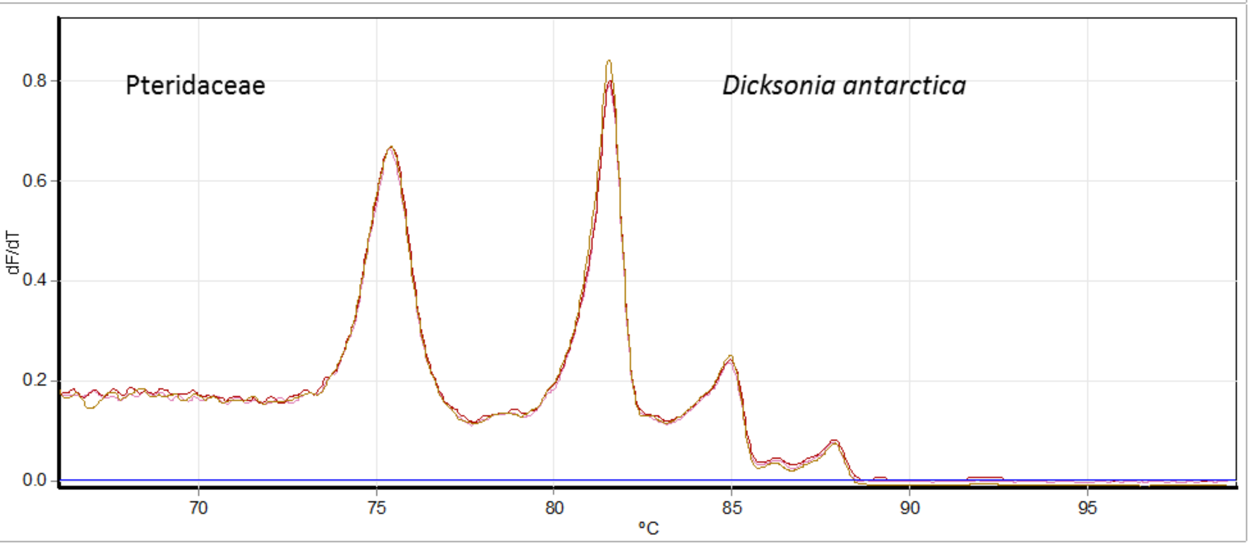
Pteridaceae
Dicksonia antarctica
dF/dT
0.8
0.6
0.4
0.2
0.0
70
75
80
85
90
95
°C

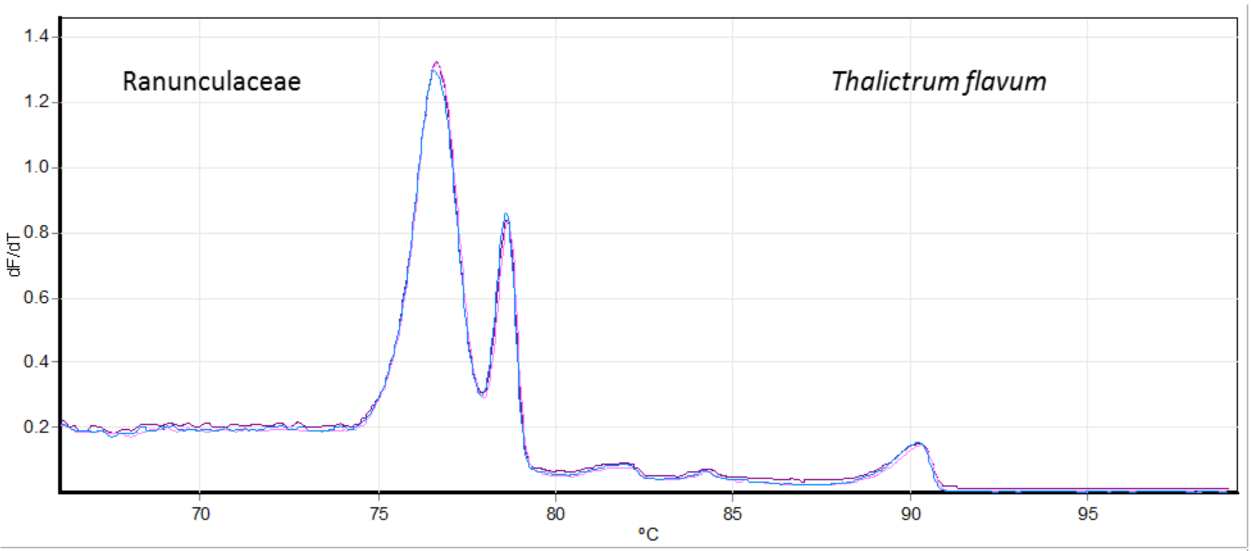
Ranunculaceae
Thalictrum flavum
dF/dT
°C
1.4
1.2
1.0
0.8
0.6
0.4
0.2
70
75
80
85
90
95

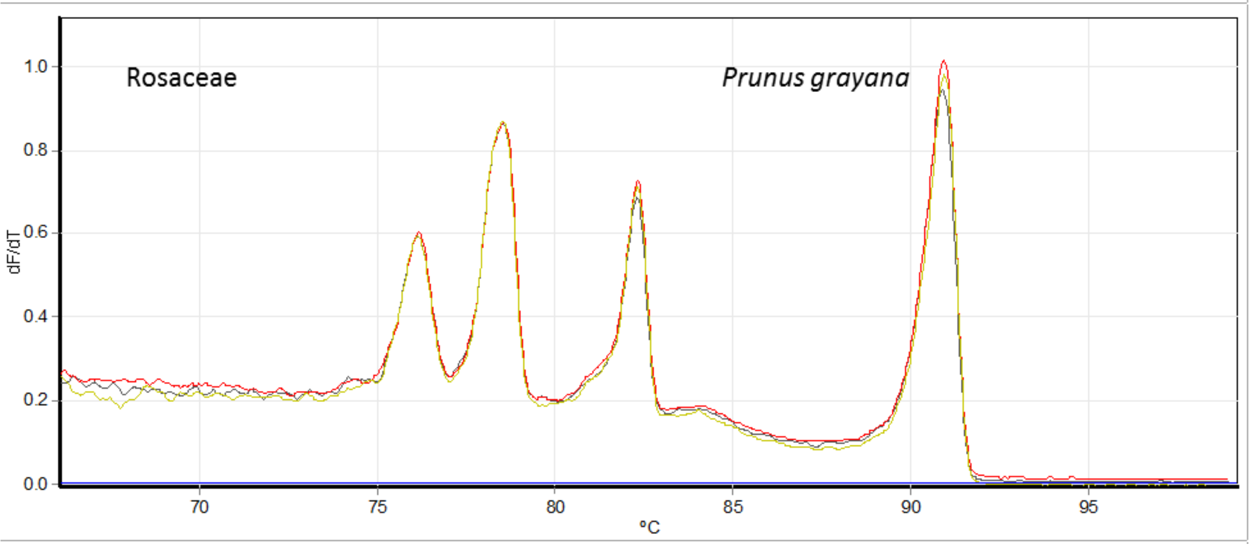
Rosaceae
Prunus grayana
dF/dT
°C
1.0
0.8
0.6
0.4
0.2
0.0
70
75
80
85
90
95

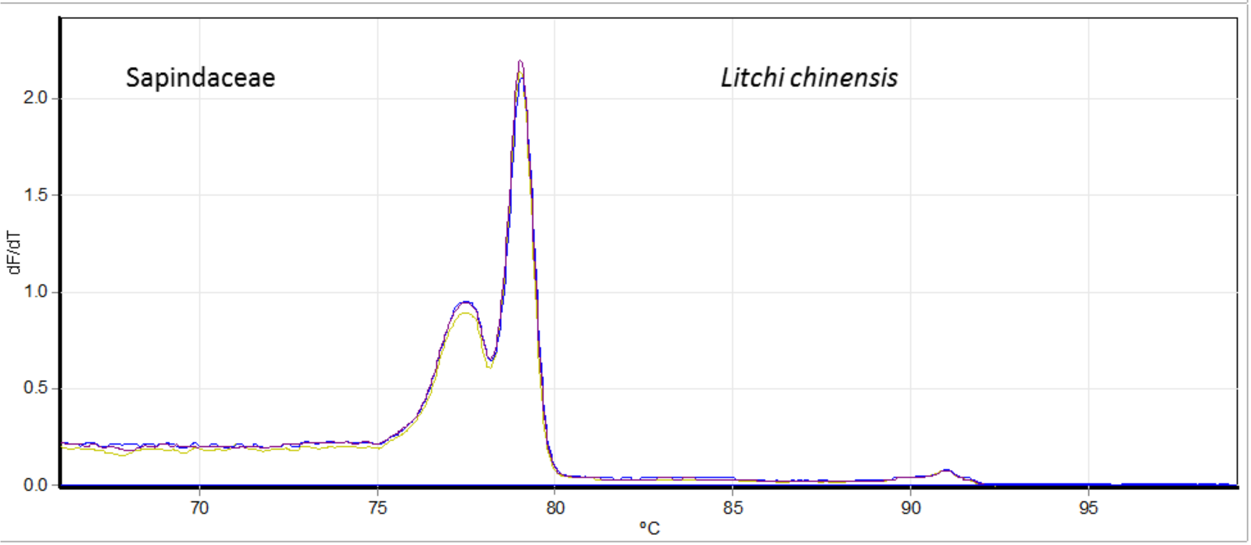
Sapindaceae
Litchi chinensis
dF/dT
°C
2.0
1.5
1.0
0.5
0.0
70
75
80
85
90
95

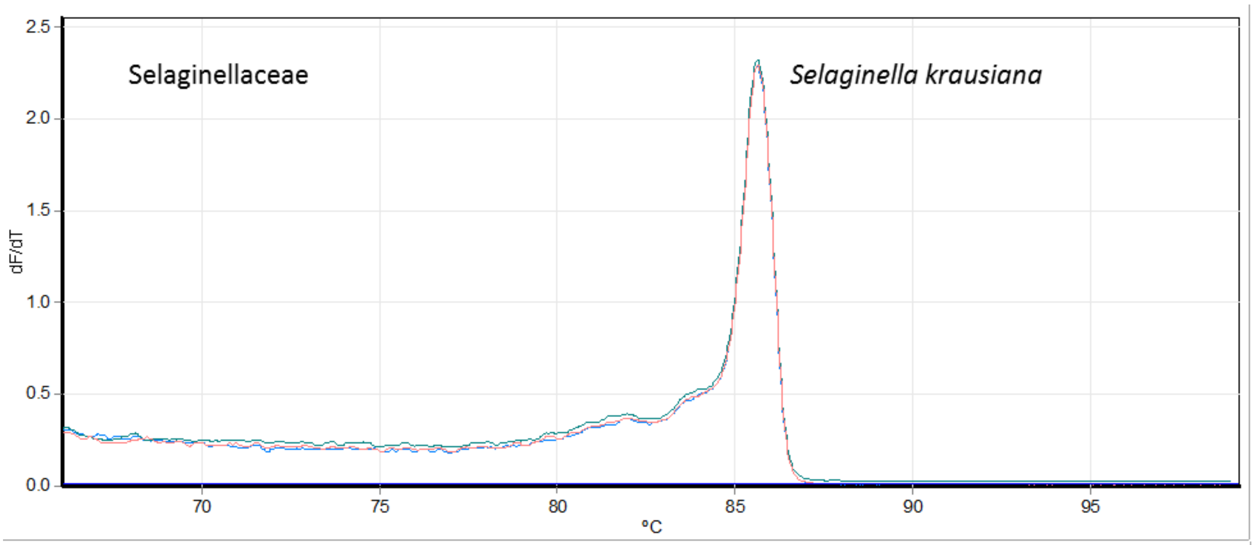
Selaginellaceae
Selaginella krausiana
dF/dT
2.5
2.0
1.5
1.0
0.5
0.0
70
75
80
85
90
95
°C

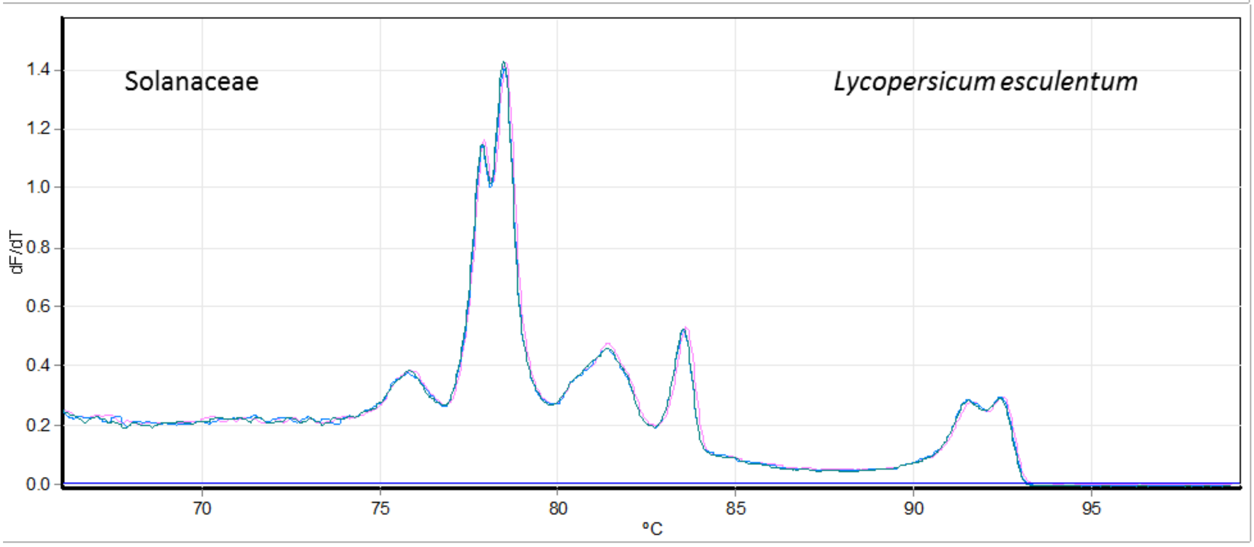
Solanaceae
Lycopersicum esculentum
dF/dT
1.4
1.2
1.0
0.8
0.6
0.4
0.2
0.0
70
75
80
85
90
95
°C
