# Supplementary material for: High-resolution melting of multiple barcode amplicons for plant species authentication
Source: Food Control. 2019 Nov;105:141–50. doi: 10.1016/j.foodcont.2019.05.022 (PMC6686639; doi:10.1016/j.foodcont.2019.05.022)
Supplement: Multimedia component 5 [file mmc5.pdf]

Supplementary 5. Repeatability, reproducibility, and intra-population variability of multiplexed melting profiles. Statistical parameters including replicate size (n), maximum (max), minimum (min), average, and standard deviation based on a sample (std (s)) were calculated in Excel and shown in each figure.

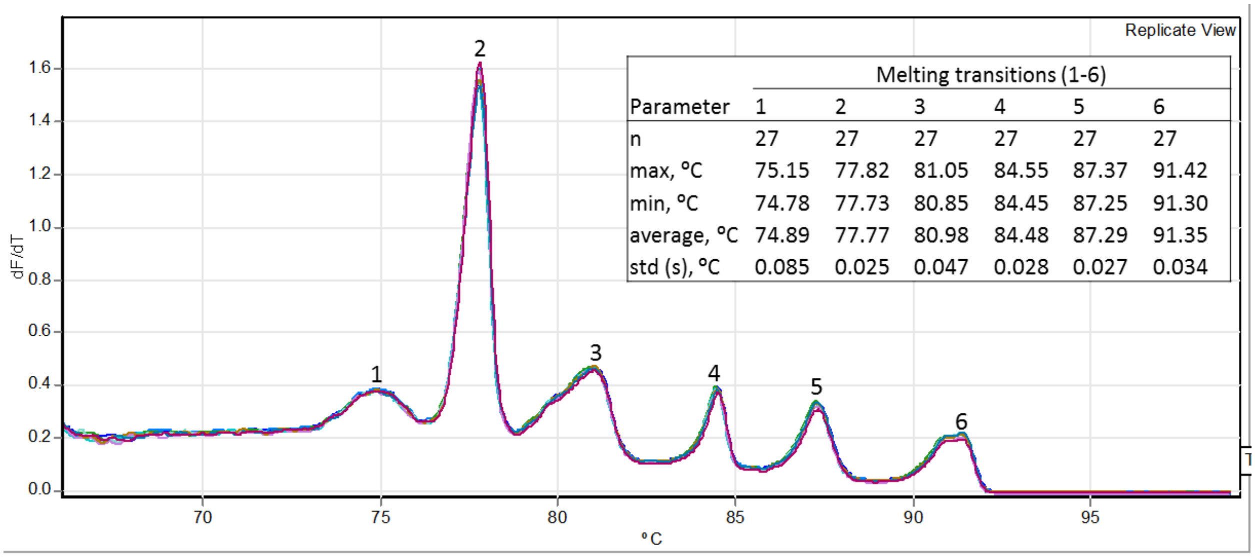

	Melting transitions (1-6)					
Parameter	1	2	3	4	5	6
n	27	27	27	27	27	27
max, °C	75.15	77.82	81.05	84.55	87.37	91.42
min, °C	74.78	77.73	80.85	84.45	87.25	91.30
average, °C	74.89	77.77	80.98	84.48	87.29	91.35
std (s), °C	0.085	0.025	0.047	0.028	0.027	0.034

Fig. 1. Repeatability. Multiplexed melting of DNA from nine *Thymus vulgaris* samples extracted the same day and analyzed in triplicate in one analytical series. This study is referred to as Experiment 1 in the article. The average of each triplicate is shown.

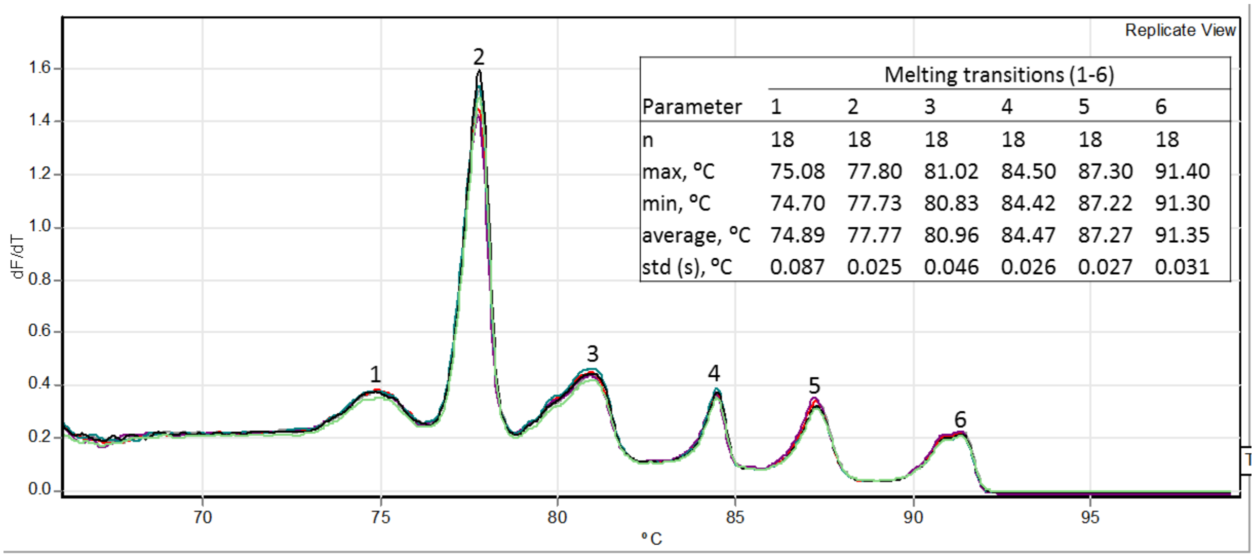

	Melting transitions (1-6)					
Parameter	1	2	3	4	5	6
n	18	18	18	18	18	18
max, °C	75.08	77.80	81.02	84.50	87.30	91.40
min, °C	74.70	77.73	80.83	84.42	87.22	91.30
average, °C	74.89	77.77	80.96	84.47	87.27	91.35
std (s), °C	0.087	0.025	0.046	0.026	0.027	0.031

Fig. 2. Reproducibility of extraction reflected on the melting profiles. Multiplexed melting of DNA from six *Thymus vulgaris* samples extracted on different days and analyzed in triplicate in one analytical series. This study is referred to as Experiment 2 in the article. The average of each triplicate is shown.

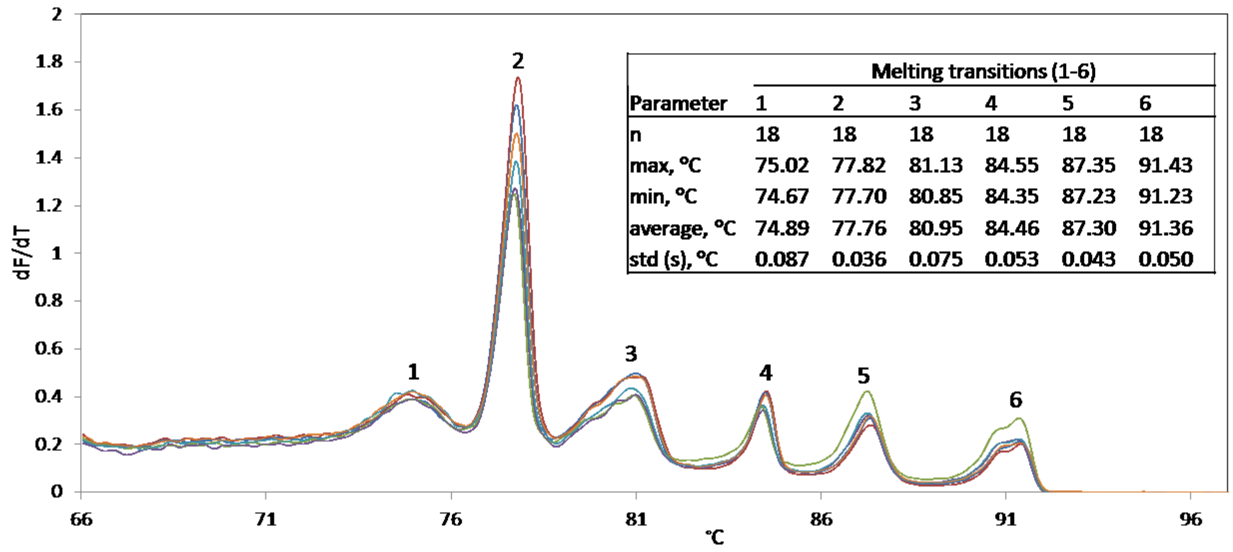

	Melting transitions (1-6)					
Parameter	1	2	3	4	5	6
n	18	18	18	18	18	18
max, °C	75.02	77.82	81.13	84.55	87.35	91.43
min, °C	74.67	77.70	80.85	84.35	87.23	91.23
average, °C	74.89	77.76	80.95	84.46	87.30	91.36
std (s), °C	0.087	0.036	0.075	0.053	0.043	0.050

Fig. 3. Reproducibility including extraction and analytical series. Multiplexed melting of DNA from six *Thymus vulgaris* samples extracted on different days and analyzed in triplicate on different days. This study is referred to as Experiment 3 in the article. The Rotor-Gene Q software cannot perform overlay from different analytical series. This figure was therefore made in Excel. The average of each triplicate is shown.

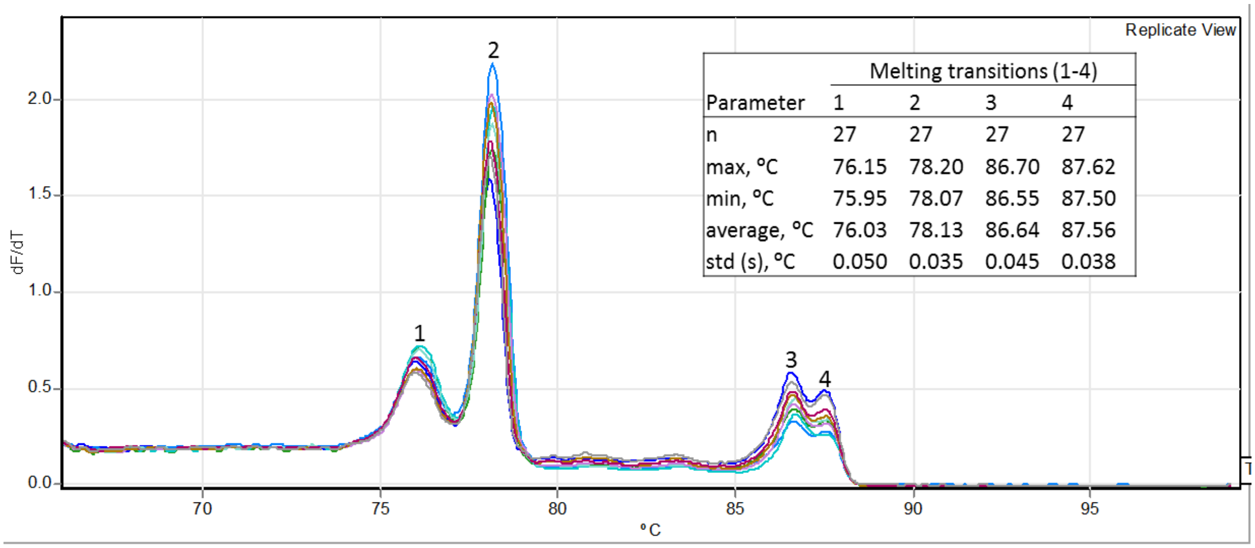

	Melting transitions (1-4)			
Parameter	1	2	3	4
n	27	27	27	27
max, °C	76.15	78.20	86.70	87.62
min, °C	75.95	78.07	86.55	87.50
average, °C	76.03	78.13	86.64	87.56
std (s), °C	0.050	0.035	0.045	0.038

Fig. 4. Intra-population variability. Multiplexed melting of DNA from nine leaves from nine plants of *Eupatorium cannabinum* extracted on the same day and analyzed in triplicate in one analytical series. The average of each triplicate is shown.
